# Development and validation of two questionnaires: Dental home care and dental health in Swedish dogs

**DOI:** 10.1371/journal.pone.0204581

**Published:** 2019-01-25

**Authors:** Karolina Brunius Enlund, Carl Brunius, Jeanette Hanson, Ragnvi Hagman, Odd Viking Höglund, Pia Gustås, Ann Pettersson

**Affiliations:** 1 Department of Clinical Sciences, Swedish University of Agricultural Sciences, Uppsala, Sweden; 2 Anicura Albano Animal Hospital, Stockholm, Sweden; 3 Department of Biology and Biological Engineering, Food and Nutrition Science, Chalmers University of Technology, Gothenburg, Sweden; Universidade do Porto Instituto de Biologia Molecular e Celular, PORTUGAL

## Abstract

**Background:**

Dental disease is one of the most common health problems in dogs. However, no studies have investigated the attitudes, opinions and practices of dog owners, veterinarians and veterinary nurses regarding dental health and preventative dental home care in dogs. The objective of this study was therefore to develop and validate questionnaires for this purpose, in accordance with survey methodology guidelines.

**Methods:**

Questionnaire items were determined based on the authors’ academic knowledge and clinical experience, and modified throughout the validation process. Several measures were taken to reduce sampling, coverage, measurement and non-response errors. Content validity was assessed by Subject-Matter Experts (SME) and cognitive interviews were conducted in accordance with the “think-aloud protocol”. Non-response analysis was performed using several methods.

**Results:**

Constructs were identified using exploratory factor analysis and two predefined constructs from the dog owner questionnaire were confirmed “Dog owners’ attitudes towards brushing their dog’s teeth” (Cronbach’s α = 0.86) and “Dog owners’ assessment of their dog’s dental health” (α = 0.76). Additionally, exploratory factor analysis identified three potential constructs. In the veterinary health practitioner questionnaire, two constructs were identified: “Veterinary health practitioners’ attitudes towards dental chews and dental feed” (α = 0.78) and “Veterinary health practitioners’ attitudes and opinions on dental problems and dental cleaning” (α = 0.73). Non-response analysis showed a higher proportion of women in the sample of dog owners and veterinarians compared to the target populations. Veterinarians in the sample were also younger. In contrast, gender and age distributions in veterinary nurses did not differ between sample and target.

**Conclusion:**

The validation presented in this work showed that the developed questionnaires could be used as accurate and reliable tools for measuring attitudes and practices regarding dental home care in dogs among Swedish dog owners, veterinarians and veterinary nurses.

## Introduction

Periodontal disease (gingivitis and periodontitis) is the most common disease affecting dogs [1], with a reported prevalence of 80–89% in dogs over 3 years of age [2–5]. Despite this high prevalence, the disease is generally considered to be underdiagnosed and therefore undertreated [6]. Periodontal disease is characterised by an inflammatory chronic loss of dental supportive tissues which may eventually result in tooth loss [4]. Studies have shown a positive correlation between severity of the disease and age, and that dogs of smaller size generally develop periodontal disease at an earlier age compared to dogs of larger size [2, 7, 8]. Daily tooth brushing is considered the gold standard both for prevention of occurrence and progression of periodontal disease [9–13]. However, tooth brushing requires good owner compliance. Today, there is a lack of studies regarding both owner compliance and the best way to motivate owners to perform oral preventative home care in dogs. It is therefore essential to investigate how dog owners receive and implement information concerning dental home care for dogs, in order to develop validated methods for optimal advice strategies to improve canine dental health. Further, it is also imperative to investigate dog owners’, veterinarians’ and veterinary nurses’ (RVT) more general attitudes, opinions and practices regarding dental health and preventative dental home care in dogs. As a way of investigating attitudes and practices regarding a specific topic, the questionnaire survey is a well-proven method, provided that it is constructed and validated according to survey methodology guidelines [14, 15].

Knowledge regarding the science of survey methodology, including construction and validation of questionnaires, should be the basis for all implemented surveys but, unfortunately, this is not always the case. General guidelines for constructing questions and response alternatives as well as methods for verifying the reliability and validity of a questionnaire are provided in the literature [16–21]. The importance of survey methodology is often underestimated, perhaps even more so in the natural sciences than in the social sciences [18]. Moreover, it is imperative that a survey is validated for each specific population and language due to the differences in social context. The application of cognitive psychology on questionnaire design has shaped the conceptual framework needed to understand the field of survey methodology, e.g. by the use of Item Response Theory (IRT) in psychometrics [17, 18]. The relatively new means of conducting surveys via the Internet offers further possibilities, such as improved survey accessibility, but also challenges [20], such as survey fatigue.

No prior study has to the authors’ knowledge presented the construction and validation of a questionnaire survey regarding dental health in dogs. Therefore, the aim of this study was to develop and validate two separate web-based questionnaire surveys for this purpose.

## Methods

The study was approved by the Regional Ethical Review Board in Uppsala (Dnr 2017/035).

### Study design

In total three target groups were defined. The target group for the dog owner survey consisted of all currently (2017) registered dog owners in Sweden. For the veterinary health practitioner survey, two target groups were identified; all registered veterinarians and all registered veterinary nurses in Sweden. Because the total population of dog owners in Sweden is unknown, register data was considered an acceptable approximation of the target population. Regarding the veterinarians and veterinary nurses, the national register data was considered very accurate and the total population was supposedly equal to the target population. The selected sample frames were dog owners, veterinarians and veterinary nurses with email addresses registered with the Swedish Board of Agriculture as at 24 February 2017 (veterinary health practitioners) or 13 March 2017 (dog owners). For veterinarians, mobile telephone numbers from the same register were also used. Furthermore, for dog owners, email addresses registered with the Swedish Kennel Club (9 February 2017) were used.

The questionnaire surveys were adapted for use on personal computers, tablets and smartphones, using the web platform Netigate (Netigate AB, Stockholm, Sweden). A text message was sent to all veterinarians with a registered mobile phone number with an invitation to participate in the survey, together with a link to the questionnaire. Additionally, emails were sent with an invitation to participate in the study, together with a link to the questionnaire, to all valid email addresses. Reminders were sent to non-respondents and non-completers after 8 and 17 days. Data collection started 31 March 2017 and was completed 30 April 2017. All responses were collected anonymously and the questionnaire could only be answered once per link. If the household owned more than one dog, the respondent was asked to choose one of them and answer for that same dog throughout the survey. Details on survey administration are reported in Table 1.

**Table 1:** Details of questionnaire recipients, responses before and after reminders, as well as median response time.

	**Recipients**	Responses* **before 1st reminder**	Responses* **after 1st reminder**	Responses* **after 2nd reminder**	**Total respondents**	**Completed questionnaires**	**Median Response time**
Veterinarians	3657	409 (sms)	263	116	1161 (32%)	1022	7 m 59 s
		373 (email)					
Veterinary nurses	1650	278	254	92	624 (38%)	572	8 m 46 s
Dog owners	209 263	33 201	22 193	11 040	66 434 (32%)	61 665	10 m 17 s

*Number of registered responses (1 / respondent) before / after day of reminder includes incompletely answered questionnaires.

### Validation of questionnaires

Questionnaire items were initially formulated by the research team and were based on veterinary expertise within dentistry as well as a thorough review of the academic literature on periodontal disease and canine dental health, supervised by the author AP (so-called stage 2 specialist, Swedish official title: “Specialist in odontology in dogs and cats”).

Item formulations were adjusted according to general guidelines of survey methodology. Subsequently, a group of consulted Subject-Matter Experts (SME, n = 3), consisting of Swedish Specialists in odontology in dogs and cats or equivalent, evaluated the content validity of the questionnaires. To ensure that perspectives from prospective respondents were well represented, a reference group (n = 3), consisting of representatives from the Swedish Kennel Club, Swedish Veterinary Association and Agria Pet Insurance, was consulted. Next, the respondents’ cognition of questions and response options was evaluated through cognitive interviews, using the “think-aloud protocol” [17]. Interview subjects were recruited by convenience sampling of respective target populations with consideration of a diversity in age and gender, until no new comments were expressed (veterinarians; n = 5, dog owners; n = 5). Lastly, pilot studies on different convenience samples of respective target groups, dog owners (Uppsala Dog Daycare; n = 22) and veterinarians (Anicura Albano Animal Hospital; n = 24), were conducted.

Additional verification of face validity was conducted by examining 200 randomly sampled free text answers in the dog owner survey and classifying these into categories. In the veterinary health practitioner questionnaire, all comments (n = 169) were examined and classified.

**Fig 1:**
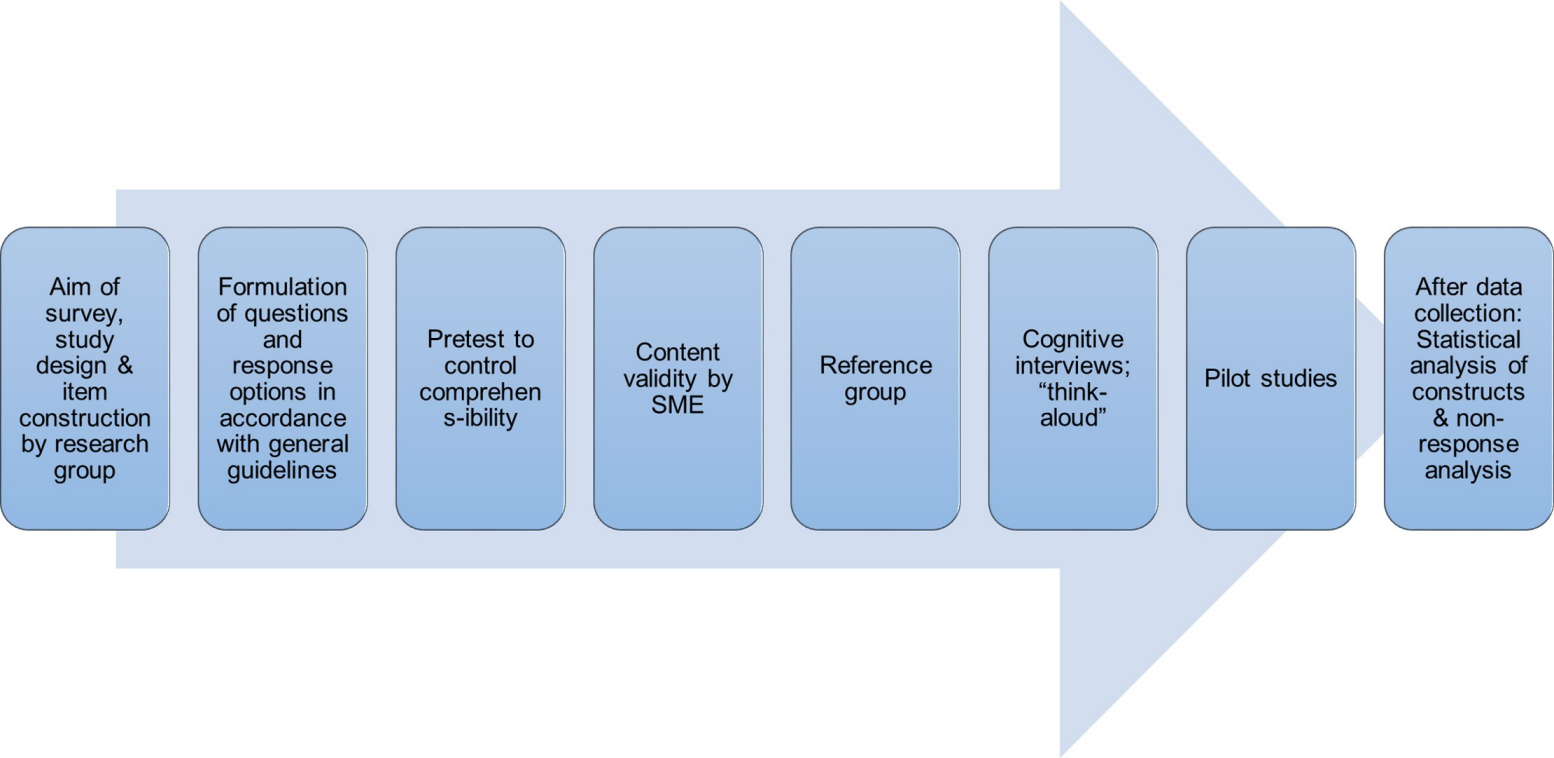
The validation process employed in the present study. Detailed information on number and order of items, construction of questions and response options are reported in the S2 Doc.

Items were adjusted and the questionnaires modified throughout the pre-defined validation process shown in Fig 1.

### Constructs

Several questions in the surveys were designed to reflect aspects of the same underlying concepts, namely 1) “Dog owners’ attitudes towards brushing their dog’s teeth” (dog owner survey), 2) “Dog owners’ assessment of their dog’s dental health” (dog owner survey) and 3) “Veterinary health practitioners’ attitudes concerning canine dental health” (veterinary health practitioner survey). The constructs were intended for use in analysis of survey results. A priori-defined constructs intended to measure these concepts were thus designed to improve measurement accuracy of the constructs through the use of multiple indicators [17]. For each construct, the authors KBE and AP therefore identified 5–6 separate questions likely to be associated with each such concept. The questions were selected based on academic knowledge and clinical experience.

### Data pretreatment

The dog owner survey originally included 66,434 respondents and 119 items with a total of 3.9 x 10^6^ missing values (49% of all data). For quantitative analyses, 4 free text variables were removed a priori. Non-completers (n = 6456) were identified as those individuals that had >20% missing values among 51 variables where missing values were less likely to occur (i.e. variables with <20% missingness). The resulting data matrix thus consisted of 59,978 respondents and 115 items (S1 Doc).

The health practitioner survey originally included 1,785 respondents (1,161 veterinarians and 624 veterinary nurses) and 84 items with a total of 66,506 missing values (44% of all data). For quantitative analyses, 5 free text variables were removed a priori. A total of 268 health practitioners (219 veterinarians and 49 veterinary nurses) who did not meet dogs in their professional life did not reply to questions regarding routines and practices and thus only answered a minor part of the questionnaire. Non-completers (n = 81; 56 veterinarians and 25 veterinary nurses) were identified as those individuals that had >20% missing values among 26 variables where missing values were less likely to occur (i.e. variables with <20% missingness). The resulting data matrix thus consisted of 1,436 respondents (886 veterinarians and 550 veterinary nurses) and 79 variables (S1 Doc).

### Non-response analysis

#### Dog owners

To assess the representativity of the sample vs the target population, non-response analyses of gender, age, geographic and dog breed distributions were performed. Information on the total population distributions was obtained from the Swedish Board of Agriculture registers. Gender distributions were compared directly using χ^2^-test. To compare age distributions, random samples (n = 1000) were drawn from the target population with sample size equal to the actual sample. Null distributions were generated from averages and standard deviations for the random samples. Two-sided tests for the sample mean and standard deviation were then performed by calculating the cumulative probability of achieving the actual average and standard deviation in the respective null distribution. Geographic distributions were compared by χ^2^-test of owners by county (län; n = 21). Quantitative tests of breed distributions were not considered due to the high number of breeds reported and because breeds were not similarly reported between sample and target populations. Qualitative comparison of the 10 most common breeds of the target and sample populations was performed.

It is generally assumed that non-respondents are more similar to late than early respondents and first vs last quartiles are therefore frequently used as a proxy for non-response analysis by assessing respondent homogeneity [22]. Due to the high number of respondents in the dog owner survey, first vs last quantiles were compared instead of quartiles for owner gender, year of birth, and response to 3 questions of topic relevance (assessment of dental health, frequency of home tooth brushing and whether dogs had undergone anaesthesia for professional dental cleaning). The quantiles were defined as the number of respondents replying to the survey after the second reminder (17.8% of completed responses) compared with 17.8% of the first responses. Comparisons were performed by two-sided t-tests except gender, which was compared by χ^2^-test.

#### Veterinary health practitioners

Non-response analyses of gender and age were performed similarly as for the dog owners, but separately for veterinarians and veterinary nurses. First and last quartiles were compared for gender, year of birth, and response to 3 questions of topic relevance (if they meet dogs in their role as professionals, how common they consider dental problems to be, and if they recommend tooth brushing for the dog) using similar tests.

#### Telephone interviews

Finally, to further assess the representativity of the sample vs the non-respondents, random samples from the target populations (n = 296 veterinarians; n = 296 dog owners) were selected for telephone interviewing (Table 2). The participating dog owners were asked 7 of the questions from the questionnaire. If they had received advice regarding dental home care for the dog from a veterinarian then they were asked 5 additional questions. In a similar manner, the veterinarians were asked 8 questions from the questionnaire. If they gave advice regarding dental homecare for dogs, they were asked 4 additional questions. Although the response rate was not sufficient for statistical analysis, results from the telephone interviews of non-respondents were compared qualitatively to the population of respondents.

**Table 2:** Telephone calls to random representatives of target populations. These were performed for qualitative assessment of non-responders, as part of the non-response analysis.

	No answer	Already answered questionnaire	Don’t work with / own dog	Declined to participate	Answered reduced questionnaire
Veterinarians (n = 296)	203 *	19	21	7	46
Dog owners (n = 296)	189	10	14	11	72

* 97 of these had international or inactive telephone numbers.

### Factor analysis

Prior to factor analyses, additional variables were removed. From the dog owner data set, 76 variables were removed (either nominal variables, ordinal with responses only from a subpopulation due to logical conditions from previous questions, with near zero variance or sociodemographic/background questions; S1 Table). From the health practitioner data set, 53 variables were removed (similar reasons as above; S1 Table). “I don’t know” or “Other” responses were substituted with either a fixed or missing value on a per-variable basis (S1 Table). Random forest imputation of missing values was performed using an in-house R script. For the dog owner data set, a total of 115,255 entries corresponding to 3.9% of data from 59,978 respondents and 47 variables were imputed. For the health practitioner data set, 223 entries corresponding to 0.6% of data from 165 respondents and 8 variables were imputed. Random forest imputation, which was used in this study, is an emerging technique that has been shown to be less conservative than fixed value imputations (e.g. mean or median imputation) and also provide more unbiased estimates compared to other dynamic techniques such as kNN [23].

The datasets was randomly split into halves and exploratory factor analysis (EFA) was performed on one half of the data to identify factors using the R ‘psych’ package v 1.7.8 [24]. Modelling was performed with maximum likelihood factoring and oblique “oblimin” rotation. For the dog owner EFA, VSS and MAP criteria for optimal number of factors showed inconsistent results and EFA was therefore performed on 3 to 6 factors. For the veterinary health practitioner EFA, both VSS and MAP criteria suggested 2 factors.

A loading cutoff of 0.4 was used to identify potential constructs from the factors obtained. To test these potential constructs, confirmatory factor analysis (CFA) was then performed on the other half of the data using the R ‘lavaan’ package v 0.5–23.1097 [25]. Modelling was performed with maximum likelihood factoring. The random split EFA/CFA procedure was repeated 10 times to investigate consistency of potential constructs. Parameter estimates and fitness measures are reported as mean ± sd for the 10 repetitions.

Identified factors were then manually inspected for relevance and compared to the pre-determined potential constructs identified by authors AP and KBE. All data pretreatment and statistical analyses were performed in the open source statistical environment R v 3.4.1 [26]. All scripts are available from the authors upon request.

## Results

### Validation process

The validation process is illustrated in Fig 1. The questionnaires were shown to have a high content validity as assessed by SME. Furthermore, high face validity was established by pre-testing, reference group consultation, cognitive interviews, pilot studies and free text analysis of the completed questionnaires.

In total, 2% of dog owners (4 of 200 random answers) and 1% (2 of 169 answers) of veterinary health practitioners expressed some sort of dissatisfaction in the free text of the surveys, either with a particular question or with the survey in general (e.g. that the questionnaire was too long).

### Questionnaire construction

In the veterinary health practitioner questionnaire, there was a final number of 49 survey questions, which were subdivided into a total of 84 questions when including multiple-choice options. In the dog owner questionnaire, there were 68 questions, subdivided into 119 questions including multiple-choice options.

In the free text analysis of the full-scale surveys one potential measurement error could be detected. Among veterinarians who had answered that they meet dogs “occasionally” within their profession (n = 245), comments (n = 18) indicated that the following questions were partly not applicable or were irrelevant to their work.

### Respondents

At the time of the survey, the target populations consisted of 607,610 registered dog owners (396,092 women and 211,518 men), 4,081 veterinarians and 1,814 veterinary nurses. Of the dog owners, 23% owned more than one dog. Details on recipients, respondents and non-response are shown in Table 1. 66% of veterinary health practitioners answered the survey by tablet or smartphone, 34% answered by desktop computer. A total of 56% of dog owners answered the survey by tablet or smartphone, 44% answered by desktop computer.

### Response rate

The reported response rates were 32% for dog owners and veterinarians, and 38% for veterinary nurses (Table 1). Median response time in the pilot studies was 10.0 minutes for the veterinary health practitioner questionnaire and 15.0 minutes for the dog owner questionnaire.

### Non-response analysis

The internal loss was ≤0.9% in the dog owner questionnaire and ≤1.7% in the veterinary health practitioner questionnaire on any single question.

**Fig 2:**
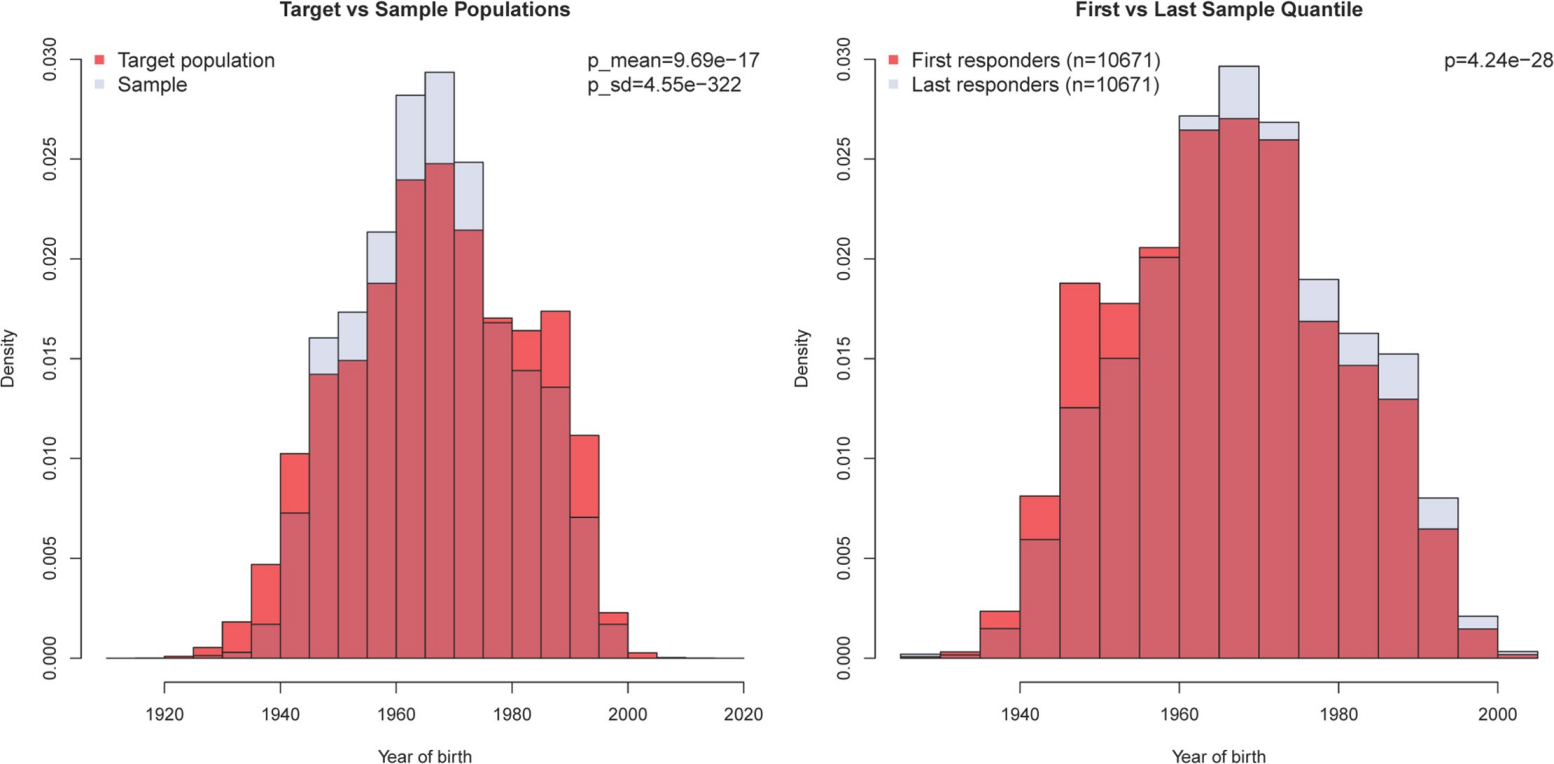
Age distribution in target and sample populations. Age distribution in target (dog owners registered in the Swedish Board of Agricultures register) and sample populations of dog owners (left) and first vs last quantiles of the sample (right). The sample population had a narrower age distribution than the target population and the last responders were systematically younger than the first responders.

The gender distribution among dog owners was significantly different between the target and sample populations (65.2% vs 74.3% women; p<0.0001). There was also a gender difference between the first and last quantiles (71.4% vs 75.2% women; p<0.0001). Mean ages of target and sample populations of dog owners were different, but not likely to be relevant because of the small effect size (0.5 years) (Fig 2). First and last quantile of dog owner respondents also differed regarding year of birth, where the last quantile was systematically younger (Fig 2). The geographic distribution by county differed between the target and sample distributions of dog owners (p<0.0001). Among the 10 most common breeds in the sample and target populations, 8 were identical and the remaining 2 breeds were found among the top 20 breeds of the other population. Of the three questions of topic relevance to the dog owners, the two questions on assessment of dental health and frequency of tooth brushing were significantly different (p<0.01) between quantiles. The third question, i.e. whether dogs had undergone anaesthesia for professional dental cleaning, did not differ between groups.

Among veterinary health practitioners, there was a higher proportion of women in the sample compared to the target population, 68.3% vs 79.6% for veterinarians, p<0.0001. The veterinarians in the sample were also younger (average 6 year difference, p<0.0001). In contrast, gender and age distributions among the veterinary nurses did not differ between target and sample population. In the two separate quartile analyses of veterinarians and veterinary nurses respectively, no significant differences were seen between first and last quartile regarding gender, age or the three selected questions from the questionnaire.

### Factor analysis

**Fig 3:**
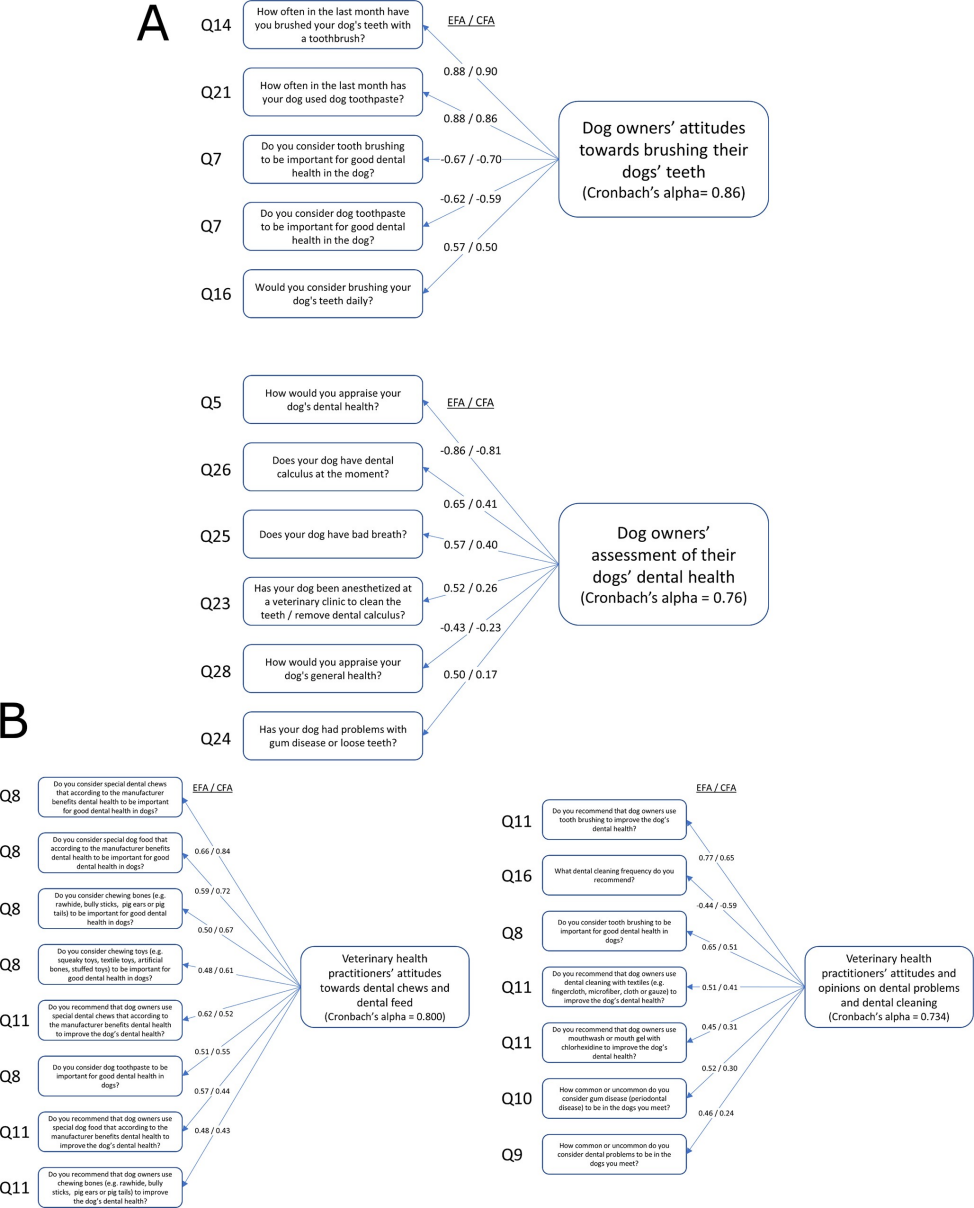
a. Factor Analysis of the dog owner questionnaire identified two constructs. Exploratory Factor Analysis (EFA) was performed on half the data to identify factors which were tested by Confirmatory Factor Analysis (CFA) on the other half. Of the five identified factors, the first two were identified as potential constructs. These constructs were named “Dog owners’ attitudes towards brushing their dogs’ teeth” (*BrushAttitude*) and “Dog owners’ assessment of their dogs’ dental health” (*DentalHealth*) and coincided with constructs predefined by the authors KBE and AP. Variable loadings are shown for both EFA and CFA. Detailed information on variables and variable/factor (co)-variance is available in S2 Table. 3b. Factor Analysis of the veterinary health practitioner questionnaire identified two constructs. Exploratory Factor Analysis (EFA) was performed on half the data to identify factors which were tested by Confirmatory Factor Analysis (CFA) on the other half, resulting in two potential constructs. These constructs were named “Veterinary health practitioners’ attitudes towards dental chews and dental feed” (*ChewFeed*) and “Veterinary health practitioners’ attitudes and opinions on dental problems and dental cleaning” (*Cleaning*). Variable loadings are shown for both EFA and CFA. Detailed information on variables and variable/factor (co)-variance is available in S2 Table.

For the dog owner survey, both the 4-factor and 5-factor models produced consistent factor identifications in the repeated-split EFA/CFA procedure. Conversely, both the 3-factor and 6-factor models produced inconsistent factor identification between repetitions and were therefore not investigated further. All factors in the 4-factor model were identically present in the 5-factor solution. Moreover, the 4-factor and 5-factor models provided similar CFA fitness metrics, but with slightly better overall fitness for the 5-factor model. Some, but not all, criteria for standard CFA fitness measures were met [27], which indicated relevant modelling results (Table 3). The 5-factor model, representing five potential constructs, was therefore chosen as the best representation of the quantitative data (S2 Table). Of the five potential constructs, three were disregarded upon manual inspection: Factor 3 was deemed too naïve, since it represented only two highly related questions (importance and frequency of use of dental sticks, respectively), whereas CFA loadings for factors 4–5 were <0.4 for all variables, suggesting weak associations between construct and variables. The two constructs identified from the remaining factors were named “Dog owners’ attitudes towards brushing dogs’ teeth” (*BrushAttitude*) and “Dog owners’ assessment of their dogs’ dental health” (*DentalHealth*), according to commonalities between underlying variables, and coincided with constructs predefined by the authors KBE and AP. (Fig 3). Analysis of internal consistency, as measured by Cronbach’s alpha, also strengthened the relatively higher reliability of the first two factors/constructs (“*BrushAttitude*” and “*DentalHealth*”) compared to the three other ones (Table 3). It should be noted that Cronbach’s alpha measures may not be entirely reliable since the assumption of tau-equivalence was not met (p<0.001).

**Table 3:** Fitness characteristics of factor solutions obtained from Dog Owners (n = 59978) and Veterinary Health Practitioners (n = 1436) surveys. The data was randomly split in halves: Exploratory Factor Analysis was performed on one half to identify factors and tested on the other half by Confirmatory Factor Analysis. The random split was repeated 10 times to further investigate the stability of identified factors. Values are reported as mean ± standard deviation for the 10 random splits.

	p-value (χ^2^)	GFI^a^	CFI^b^	RMSEA^c^	SRMR^d^	α^e^ F1	α F2	α F3	α F4	α F5
Dog Owners	<0.001	0.932 ± 0.001	0.879 ± 0.001	0.069 ± 0.000	0.046 ± 0.000	0.863 ± 0.001	0.759 ± 0.002	0.746 ± 0.001	0.642 ± 0.003	0.638 ± 0.003
Veterinary Health Practitioners	<0.001	0.815 ± 0.012	0.633 ± 0.028	0.127 ± 0.005	0.115 ± 0.007	0.776 ± 0.011	0.734 ± 0.007	NA	NA	NA

^a^ Goodness of Fit Index

^b^ Comparative Fit Index

^c^ Root Mean Square Error of Approximation

^d^ Standardized Root Mean Square Residual

^e^ Cronbach’s alpha per factor

For the veterinary health practitioner survey, consistent factor identifications in the repeated-split EFA/CFA procedure was achieved for 2 factors as suggested by both VSS and MAP criteria. These potential constructs were named “Veterinary health practitioners’ attitudes towards dental chews and dental feed” (*ChewFeed*) and “Veterinary health practitioners’ attitudes and opinions on dental problems and dental cleaning” (*Cleaning*), according to commonalities between underlying variables (Fig 3B). A sensitivity analysis of the factoring was performed by investigating also 1-factor and 3-factor models. The 1-factor solution was identical to the first factor of the 2-factor model, but had worse CFA fitness (data not shown), whereas the 3-factor model produced inconsistent factor identification between repetitions, indicating a poorer fit to the data. Both constructs showed adequate internal consistency as measured by Cronbach’s alpha (α > 0.73), with the caveat that the tau-equivalence assumption was not met (p<0.001) and that alpha estimates may not be entirely reliable. However, CFA fitness metrics indicated a poorer fit to the data compared to the dog owner survey (Table 3).

## Discussion

The importance of a well-constructed and validated questionnaire should not be underestimated, and survey methodology should always be applied when working with questionnaires in research, regardless of orientation or topic, to assure high quality of measured data.

Two questionnaire surveys about attitudes and practices regarding dental health in dogs have previously been published: One study was directed to Dutch veterinarians in 1991 and the other to veterinarians and veterinary nurses in the UK in 2012. However, unlike the present study, none of these presented any validation of the questionnaires [28, 29]. To the authors’ knowledge, this is the first study presenting the development and validation of a questionnaire survey regarding dental health in dogs.

### Validation process

The validation protocol used in this study was developed and conducted according to current evidence-based recommendations taking into account several aspects of questionnaire validity (Fig 1) [14, 16, 17, 30]. Reference group consultation and cognitive interviews generated comments, which resulted in revision of the questionnaires to increase clarity and simplicity. Similarly, pilot studies on target groups raised comments and concerns, which resulted in further questionnaire revision. The questionnaires were revised continually throughout the validation procedure. Pre-testing of the questionnaires, together with cognitive interviews and discussions with the Subject-Matter Experts and the reference group, showed high questionnaire comprehensibility. In addition, the questionnaires were shown to cover the topic in accordance with the aims of the study, thus indicating high content as well as face validity. The free text analysis showed a low degree of respondent discontent and no misunderstandings were detected, further indicating high face validity.

### Questionnaire construction

Some veterinarians who only met dogs occasionally experienced the questionnaire as partly irrelevant, and reported e.g. that they only performed vaccinations, were retired or only worked administratively. This could potentially induce measurement errors. For instance, these veterinarians could answer either “no, never” or “don’t know” on the question of whether they recommend tooth brushing for the dog. However, our research hypothesis was that even veterinarians that do not meet dogs regularly may influence dog owners regarding dental care. Thus, we strived to reach a wider range of veterinarians, not only small veterinary practitioners, and these differences in employment are taken into account when analysing data. After requests in the “think-aloud” interviews, the option “sometimes important” was added as a response option in addition to non-frequency directed options, in the question about what veterinary health practitioners consider to be important for good dental health in dogs. However, mixing response scales may cause difficulties in interpreting results.

Through qualitative analysis of the free text comments in the full survey, additional issues were identified, which were not detected during the validation procedure. One question asked about when the veterinary health practitioners informed dog owners about tooth brushing. However, it was not possible to make any distinction between giving information for pre-existing dental problems, or if information was provided as preventative education (to all patients / subgroups). In addition, it was not possible to discriminate if the provided recommendations differed depending on breed.

In the dog owner questionnaire, hundreds of dog owners commented that a question regarding number of dogs owned by each respondent was missing. In fact, as many as 23% of Swedish dog owners own more than one dog (Personal Communication, Magnus Kindström, Swedish Board of Agriculture, 28 August 2017). Although the respondents were asked in the introductory letter to “answer just for one dog only” in case they owned more than one dog, it seems that many owners failed to note this information. In retrospect, this information could have been given on more occasions to avoid unnecessary confusion. However, in the full study, additional comments were limited to a few questions, with consequently limited negative impact for the study as a whole. It can thus be concluded that the thorough construction procedure resulted in two final questionnaire formulations of high quality, thereby providing useful tools with which to collect the desired data concerning dental health in dogs.

### Respondents and response rate

The decision to use an electronic questionnaire may have resulted in the exclusion of respondents without access to a computer or smartphone, and also respondents that had not reported an e-mail address to the registers. This may possibly lead to a sample error, with fewer older respondents as well as fewer older dogs in the survey since the use of e-mail may have increased during the last 10 years. In 2017, a Swedish national survey estimated the number of Internet users at between 92% and 100% of the total population between 12–75 years of age. However, among people over 76 years old, the number of Internet users decreased to 56%, which may partly explain the relatively low number of respondents in this group [31]. On the other hand, the advantage of electronic distribution lies in the rapid, cost-effective means by which many respondents can be reached. The fact that more than half of the respondents used tablet or smartphone shows the importance of adapting the questionnaire template to different technical platforms, which in the present study was provided by the web platform Netigate. Other distribution methods, e.g. links in social media, were disregarded because of the presumed decrease in representativity (recruitment bias), e.g. people with a greater interest in the topic of the survey tend to participate to a higher degree.

In this study, because of over-coverage (e.g. if a person is no longer a dog owner) and under-coverage (e.g. due to inaccurate email addresses or computer security settings) the actual sample frame most likely differed from the number of email addresses and mobile phone numbers used, with potential coverage error as a consequence [14]. Although it is not possible to know exactly how many invitations did not reach the intended recipient, over-coverage may have resulted in a higher number of younger and / or newer respondents, both among dog owners and veterinary health practitioners. Consequently, the reported response rates (Table 1) are presumed to be underestimated.

Reported response rates for electronic questionnaires vary widely and no consensus on what constitutes a normal response rate seems to exist. In fact, Couper and Bosnjak report response rates from 3% up to 77% [14], with the higher proportion derived from a web-based panel of respondents and the lower from using pop-up questions upon visiting a website. Unfortunately, the vast number of customer surveys, polls and research surveys have resulted in decreased questionnaire response rates because of survey fatigue [14] and what can be perceived as an acceptable response rate differs widely between authors and has changed over time. Fifty years ago, a response rate of at least 70% was considered absolutely necessary [20], whereas today, such a response rate must be regarded as high. Moreover, a meta-analysis made in 2008 showed that electronic questionnaires on average have an 11% lower response rate than postal questionnaires [32]. However, response rates in the present study were similar to those of a postal questionnaire survey in 2012 investigating the perception of canine dental health among veterinarians and veterinary nurses in the UK [29].

The most important aspects to consider in order to achieve a high response rate are clear, logical, well-devised item formulations, short survey length and an interesting topic [14, 16]. Shorter questionnaires usually gain higher response rates [33, 34] and this study therefore aimed for a completion time of less than 10–15 minutes for the majority of respondents. During the construction of the questionnaires, the number of questions was adjusted to minimise respondent burden. The pilot studies showed a median response time of between 10 minutes (veterinary health practitioners) and 15 minutes (dog owners) which was regarded as a sufficiently short questionnaire. Response rates are also affected by whether respondents find the topic emotionally engaging [22, 34]. Dog owners may be expected to find questions about their dog easy and enjoyable to answer and therefore these questions were placed first in the questionnaire [14, 35].

To further increase response rates, rewards may sometimes be given upon completion. However, the data on the use of rewards is somewhat ambiguous [34] and also since the topic of this study was assumed to be strongly engaging, especially for dog owners, we chose not to apply this strategy. Other factors such as day and time of distribution, reminders, and the visual appearance (the lay-out) of the questionnaire may also contribute to response rates [34, 36]. Some survey-generators suggest better response rates if the questionnaire is sent out early in the week, therefore this approach was used in the present study. However the data on this is also ambiguous [34]. Distributing the survey over public holidays can decrease response rates, which is why this was avoided [16]. The subject of the questionnaire is likely to affect the optimal time for send-out, whether it is work-related (veterinary health practitioners) or not (dog owners). Reminders were sent out after a little more than one and two weeks to non-completers [20], and the general recommendation that there should not be more than two reminders [16] was also followed. However, Deutskens et al. reported that the timing of reminders does not have a significant impact on response rate [33].

### Non-response analysis

Internal loss, e.g. caused by technical errors or interruptions, was observed to be mainly small-scale and random and was managed by imputation to avoid excluding observations and consequent loss of power.

#### Dog owners

The observed differences between sample and target population corresponded to what is frequently reported in the literature, i.e. older people are more likely to respond than younger; women respond to a higher degree than men, and those with higher education are more likely to respond than those with lower education. This coincides with a higher number of respondents from urban areas, where a higher general level of education can be observed [37, 38]. The geographic distribution of dog owners and the distribution of dog breeds indicated high representativity of respondents to the target population.

Contrary to the general notion that late respondents have more in common with non-respondents than do the early responders [22], the last quantile did not show a higher similarity with non-respondents with regard to gender in this survey. Moreover, the difference in responses to the questions on assessment of dental health and frequency of tooth brushing between first and last quantiles was very small (in fact only ≈1% effect size difference between groups), indicating homogeneity among respondents.

#### Veterinary health practitioners

Gender and age distributions in veterinary nurses did not differ between target and sample, likely due to the predominance of women (96.7%) and younger ages in the target population. Among veterinarians, however, gender and age distributions differed between target and sample similar to the dog owners, which reflects a possible source of bias. However, no differences were observed in the quartile analyses, indicating homogeneity among respondents and high representativity of respondents to the target population.

Telephone interviews with non-respondents were conducted as an alternative approach to qualitatively assess possible differences from respondents (Table 2). Social desirability bias is likely to have a larger influence in a telephone interview situation than in a web-based survey, which may at least partly explain that the telephoned dog owners reported brushing their dogs’ teeth to a greater extent compared to the survey respondents. The telephoned veterinarians were less prone to recommend tooth brushing for the dog, which may reflect the fact that non-respondents have been shown to show less interest than respondents in the questionnaire topic [34].

To conclude, the analysis agreed with the aforementioned trends observed in questionnaire surveys in western countries, which may affect the representativity of respondents to the total population. However, given the homogeneity and small effect size of the differences we consider the respondents to be representative of our target population.

### Factor analysis

Factor analysis (FA) can greatly help reduce complexity in data analysis by both reducing the rank of the original data and improving signal-to-noise, by combining several variables into constructs. FA was performed with the primary aim to provide tools to be able to investigate attitudes, opinions and practices regarding dental home care in dogs in the collected study material. Secondary aims included to be able to use identified constructs for longitudinal national follow-up, as well as for international comparisons.

In addition to the CFA fitness metrics of the five factors identified in the dog owner EFA (Table 3), the fact that the two strongest factors (“*BrushAttitude*” and “*DentalHealth*”) corresponded to the two constructs that were a priori-determined by the authors KBE and AP provided additional confirmation of these constructs through in-field competence (Fig 3A). The three remaining factors from the EFA had not been previously identified and thus potentially indicated additional, unforeseen constructs. However, these potential constructs were disregarded upon manual inspection of variables selected and estimate variability (S2 Table), regardless of CFA fitness metrics (Table 3).

In the veterinary health practitioner FA, two factors were identified as potential constructs (Fig 3B). The pre-determined construct “Veterinarians’ and veterinary nurses’ attitudes concerning canine dental health” was not observed to qualitatively correspond directly to the two factors, possibly at least partly since only quantitative variables were used and much of the information on attitudes therefore were omitted from the FA. The two factors instead seemed to reflect more detailed aspects of the pre-determined construct (Fig 3B). Sensitivity analysis, conducted as FA with only one factor, also did not correspond to the pre-determined factor, but was instead a perfect fit for the “*ChewFeed*” construct. Although Chronbach’s alpha suggested adequate internal consistency for both factors, the comparably low CFA fit of the Veterinary Health Practitioner FA model to the data suggests that these constructs may not be accurate or even applicable and should be used, if at all, mainly for data exploratory purposes and results interpreted with caution.

Assumptions of tau-equivalence or parallel item model were not met for either Dog Owner or Veterinary Health Practitioner FA (p<0.001), indicating that the raw scores from the survey should not be directly summed into composite scores. To investigate research questions relating to the constructs, factor scores should instead be used. Parameter estimates and variance-covariance matrices to calculate factor scores corresponding to the identified constructs are provided in S2 Table.

### Limitations

A questionnaire is only valid in the language and target population it is tested for. The presented questionnaires in this study are thus only validated for use in the Swedish language and in a Swedish social and cultural context. If they were to be used in another country, back-forward translation would be the minimum level of quality control needed and could be sufficient if the social and cultural settings were similar [14]. However, the authors would suggest minor improvements, as mentioned in the discussion.

Although a possible source of bias, precautions were taken to avoid acquiescence bias, i.e. the tendency of a respondent to agree with a statement when in doubt, by careful wording of questions. Social desirability bias can also be problematic [14], and may lead to the owners and veterinary health practitioners reporting a false high degree of awareness and / or practice of dental home care. To avoid this as far as possible no judgmental formulations were used.

Inter-rater agreement was not used to assess test reliability, since most of the data was collected on fixed scales and thus considered unaffected by assessor. Furthermore, the same individual assessed all qualitative aspects of all free text answers. Test-retest was not applied to test reliability, since the subject of the survey (i.e. dental health in dogs) was regarded as specific and therefore sensitive to repeated measurement. It was thus assumed that a second measurement would be affected by the first survey raising respondent awareness.

Criterion validity, i.e. the extent to which a measure is related to a specific outcome, was not possible to establish because the dogs were not examined clinically, nor was contact established with respondents in any other way to examine other proxy measures of outcomes (e.g. quality of performed home care or owners actual ability to correctly assess the dog´s dental health). Construct validity can sometimes be assessed by comparing results with known facts. Since it was not possible to determine reference states of respondents’ attitudes and practices, this was not possible in the present study. Furthermore, to the best of our knowledge, no study has investigated how common tooth brushing in dogs actually is, and there are no reliable figures available on the prevalence of dental disorders in dogs in Sweden.

It is always tempting to collect as much information as possible from a survey; however, it is important to keep a survey short. Further questions to dog owners on the dog’s feeding habits, and also an investigation of dog owners, veterinarians and veterinary nurses’ knowledge regarding canine dental health, are possible subjects for future studies.

## Conclusion

In the present article, we describe the development and validation process of two valid and reliable Internet-based questionnaires used to study attitudes and practices regarding dental home care in dogs among Swedish dog owners, veterinarians and veterinary nurses. In the Dog Owner survey, two constructs could be identified: “Dog owners’ attitudes towards brushing their dogs’ teeth” and “Dog owners’ assessment of their dogs’ dental health”.

## Supporting information

S1 Doc File containing translated questionnaire (English) to dog owners and translated questionnaire (English) to veterinary health practitioners. (PDF)

S2 Doc File containing detailed survey construction. (DOCX)

S3 Doc Correlation matrices of variables included in factor analyses. (XLSX)

S1 Table File containing questionnaire items in Swedish, in English, short variable names, why some items are not included in the EFA, action on “”Don’t know” and action on missing value. (XLSX)

S2 Table File containing factor analysis estimates. (XLSX)
