# Supplementary material for: Development and validation of two questionnaires: Dental home care and dental health in Swedish dogs
Source: PLoS One. 2019 Jan 25;14(1):e0204581. doi: 10.1371/journal.pone.0204581 (PMC6347148; doi:10.1371/journal.pone.0204581)
Supplement: S1 Doc — (PDF) [file pone.0204581.s001.pdf]

Sveriges lantbruksuniversitet
Swedish University of Agricultural Sciences

Welcome to our survey about canine dental health!

This survey is for those of you who have a dog. If you have more than one dog we would like you to answer just for one dog only. The survey should take about 10-15 minutes to complete and your responses are only reported at the group level. Your answers are vital to our research on dog dental health and if you choose not to answer we cannot replace you with anyone else. We are very grateful for your participation.

Kindly,
Ann Pettersson, head researcher
Associate Professor, DVM, PhD, Swedish Specialist odontology dogs and cats

Karolina Enlund
PhD student, DVM

PS: Your details were given to us by either the Swedish Kennel Club or the Swedish Board of Agriculture. We are very sorry for any distress caused if your dog has since died. Dogs are not automatically removed from any registers which is why your details were passed to us. In the event that your dog is no longer with you you may choose to ignore this survey if you wish.

Sveriges lantbruksuniversitet
Swedish University of Agricultural Sciences
Department of clinical sciences
Box 7054, 750 07 UPPSALA
Visiting address: Ulls väg 26

Tel: 018-67 10 00
tandhalsahoshund@slu.se
www.slu.se

Research Institution: Swedish University of Agricultural Sciences. Participation is voluntary and can be terminated at any time without any consequences. No unauthorized person will know how you answered.
The Swedish University of Agricultural Sciences is responsible for personal data relating to this survey. The Personal Data Act, PUL (1998: 204), states that once ayear you are entitled to receive, free of charge, details of all the information about yourself that is beeing processed and, if necessary, have any errors corrected. Contact person: Ann Pettersson, ann.pettersson@slu.se, 018-67 10 00. The project is funded by Stiftelsen Djursjukhus i Stor Stockholm and Svenska Djurskyddsföreningen.

1. What year was your dog born?

Choose a year below

- ☐ 2003 or earlier
- ☐ 2004
- ☐ 2005
- ☐ 2006
- ☐ 2007
- ☐ 2008
- ☐ 2009
- ☐ 2010
- ☐ 2011
- ☐ 2012
- ☐ 2013
- ☐ 2014
- ☐ 2015
- ☐ 2016
- ☐ 2017
- ☐ Don´t know

2. What breed is your dog?

Choose an alternative below

- ☐ Mixed breed
- ☐ Don´t know / Prefer not to answer
- ☐ AFFENPINSCHER
- ☐ AFGHAN HOUND
- ☐ ATLAS SHEPHERD DOG
- ☐ AIREDALE TERRIER
- ☐ JAPANESE AKITA
- ☐ ALASKAN MALAMUTE
- ☐ ALPINE DACHSBRACKE
- ☐ AMERICAN AKITA
- ☐ AMERICAN FOXHOUND
- ☐ AMERICAN STAFFORDSHIRE TERRIER
- ☐ AMERIKANSK COCKER SPANIEL
- ☐ ANATOLIAN SHEPHERD DOG
- ☐ APPENZELL MOUNTAIN DOG
- ☐ AUSTRALIAN CATTLE DOG
- ☐ AUSTRALIAN KELPIE
- ☐ AUSTRALIAN SHEPHERD
- ☐ AUSTRALIAN STOCK DOG/WORKING KELPIE
- ☐ AUSTRALIAN TERRIER
- ☐ AZAWAKH
- ☐ BARBET
- ☐ BASENJI
- ☐ BASSET ARTÉSIEN NORMAND
- ☐ BASSET FAUVE DE BRETAGNE
- ☐ BASSET HOUND
- ☐ BAVARIAN MOUNTAIN SCENTHOUND
- ☐ BEAGLE
- ☐ BEARDED COLLIE
- ☐ BEAUCERON
- ☐ BEDLINGTON TERRIER
- ☐ BELGIAN SHEPHERD DOG/ GROENENDAEL
- ☐ BELGIAN SHEPHERD DOG/ LAEKENOIS
- ☐ BELGIAN SHEPHERD DOG/ MALINOIS
- ☐ BELGIAN SHEPHERD DOG/ TERVUEREN
- ☐ BERGAMASCO SHEPHERD DOG
- ☐ PYRENEAN SHEEPDOG-SMOOTH FACED
- ☐ PYRENEAN SHEEPDOG-LONG-HAIRED
- ☐ BERGER PICARD
- ☐ BERNESE MOUNTAIN DOG
- ☐ BICHON FRISE

- ☐ HAVANESE
- ☐ BLACK AND TAN COONHOUND
- ☐ BLOODHOUND
- ☐ BLUETICK COONHOUND
- ☐ BOLOGNESE
- ☐ BORDER COLLIE
- ☐ BORDER TERRIER
- ☐ BORZOI
- ☐ BOSNIAN COARSE-HAIRED HOUND
- ☐ BOSTONTERRIER
- ☐ BOUVIER DES ARDENNES
- ☐ BOUVIER DES FLANDRES
- ☐ GERMAN BOXER
- ☐ BRACCO ITALIANO
- ☐ BRITTANY
- ☐ BRIARD
- ☐ BROHOLMER
- ☐ BULLMASTIFF
- ☐ BULL TERRIER
- ☐ CAIRN TERRIER
- ☐ CANAAN DOG
- ☐ CANE CORSO
- ☐ SERRA DA ESTRELA MOUNTAIN DOG, LONG-HAIRED
- ☐ CAVALIER KING CHARLES SPANIEL
- ☐ CZESLOVAKIAN WOLFDOG
- ☐ CESKY TERRIER
- ☐ POLISH GREYHOUND
- ☐ CHESAPEAKE BAY RETRIEVER
- ☐ CHIHUAHUA, SMOOTH-HAIRED
- ☐ CHIHUAHUA, LONG-HAIRED
- ☐ CHINESE CRESTED DOG
- ☐ CHODSKÝ PES
- ☐ CHOW CHOW
- ☐ URUGUAYAN CIMARRÓN
- ☐ CIRNECO DELL'ETNA
- ☐ CLUMBER SPANIEL
- ☐ ENGLISH COCKER SPANIEL
- ☐ COLLIE, SMOOTH
- ☐ COLLIE, ROUGH
- ☐ COTON DE TULÉAR
- ☐ CURLY COATED RETRIEVER
- ☐ DALMATIAN
- ☐ DANDIE DINMONT TERRIER
- ☐ DANISH-SWEDISH FARMDOG
- ☐ DOBERMANN
- ☐ DOGO ARGENTINO
- ☐ DOGO CANARIO
- ☐ DOGUE DE BORDEAUX
- ☐ DRENTSE PARTRIDGE DOG
- ☐ DREVER
- ☐ DUNKER HOUND
- ☐ MINIATURE PINSCHER
- ☐ MINIATURE SCHNAUZER, PEPPER & SALT
- ☐ MINIATURE SCHNAUZER, BLACK
- ☐ MINIATURE SCHNAUZER, BLACK AND SILVER
- ☐ MINIATURE SCHNAUZER, WHITE
- ☐ ENGLISH BULLDOGG
- ☐ ENGLISH SETTER
- ☐ ENGLISH SPRINGER SPANIEL
- ☐ ENGLISH TOY TERRIER
- ☐ ENTLEBUCH MOUNTAIN DOG
- ☐ ESTONIAN HOUND
- ☐ EURASIAN

- ☐ PHARAOH HOUND
- ☐ FIELD SPANIEL
- ☐ FILA BRASILEIRO
- ☐ FINNISH LAPPHUND
- ☐ FINSK SPITZ
- ☐ FINSK HOUND
- ☐ FLAT COATED RETRIEVER
- ☐ FRENCH BULLDOGG
- ☐ SPANISH GREYHOUND
- ☐ OLD DANISH POINTING DOG
- ☐ GOLDEN RETRIEVER
- ☐ POLISH HUNTING DOG
- ☐ GORDON SETTER
- ☐ CATALAN SHEEPDOG
- ☐ GOTLAND HOUND
- ☐ GOTLAND HOUND (RASVÅRD)
- ☐ GRAND BASSET GRIFFON VENDÉEN
- ☐ GREAT DANE
- ☐ GRAND GRIFFON VENDÉEN
- ☐ GREYHOUND
- ☐ BELGIAN GRIFFON
- ☐ BLUE GASCONY GRIFFON
- ☐ BRUSSELS GRIFFON
- ☐ FAWN BRITTANY GRIFFON
- ☐ GRIFFON NIVERNAIS
- ☐ LARGE MüNSTERLANDER
- ☐ GREAT SWISS MOUNTAIN DOG
- ☐ GREENLAND DOG
- ☐ HALDEN HOUND
- ☐ HAMILTON HOUND
- ☐ HANOVERIAN SCENTDOG
- ☐ DUTCH SHEPHERD DOG, SHORT-HAIRED
- ☐ DUTCH SHEPHERD DOG, LONG-HAIRED
- ☐ DUTCH SHEPHERD DOG, ROUGH-HAIRED
- ☐ HOVAWART
- ☐ CROATIAN SHEEPDOG
- ☐ HÄLLEFORSHUND
- ☐ IRISH GLEN OF IMAAL TERRIER
- ☐ IRISH SOFTCOATED WHEATEN TERRIER
- ☐ IRISH RED AND WHITE SETTER
- ☐ IRISH RED SETTER
- ☐ IRISH TERRIER
- ☐ IRISH WOLFHOUND
- ☐ IRISH WATER SPANIEL
- ☐ ICELANDIC SHEEPDOG
- ☐ ISTRIAN SHORT-HAIRED HOUND
- ☐ ITALIAN GREYHOUND
- ☐ JACK RUSSELL TERRIER
- ☐ JAPANESE CHIN
- ☐ JAPANESE SPITZ
- ☐ SOUTH RUSSIAN SHEPHERD DOG
- ☐ SWEDISH ELKHOUND
- ☐ KAI
- ☐ KARELIAN BEAR DOG
- ☐ CAUCASIAN SHEPHERD DOG
- ☐ KEESHOND
- ☐ KERRY BLUE TERRIER
- ☐ KING CHARLES SPANIEL
- ☐ SMALL MüNSTERLANDER
- ☐ KOMONDOR
- ☐ KOREA JINDO DOG
- ☐ GERMAN SHORT-HAIRED POINTING DOG
- ☐ KROMFOHRLÄNDER
- ☐ KUVASZ

- ☐ LABRADOR RETRIEVER
- ☐ LAGOTTO ROMAGNOLO
- ☐ LAKELAND TERRIER
- ☐ LANCASHIRE HEELER
- ☐ LANDSEER
- ☐ LAPONIAN HERDER
- ☐ LEONBERGER
- ☐ LHASA APSO
- ☐ GERMAN LONG-HAIRED POINTING DOG
- ☐ LÖWCHEN
- ☐ HUNGARIAN GREYHOUND
- ☐ MALTESE
- ☐ MANCHESTER TERRIER
- ☐ MAREMMA AND ABRUZZES SHEEPDOG
- ☐ MASTIFF
- ☐ SPANISH MASTIFF
- ☐ NEAPOLITAN MASTIFF
- ☐ MINIATURE BULL TERRIER
- ☐ PUG
- ☐ MUDI
- ☐ NEDERLANDSE KOOIKERHONDJE
- ☐ NEWFOUNDLAND
- ☐ NORFOLK TERRIER
- ☐ NORRBOTTENSPITZ
- ☐ NORWEGIAN BUHUND
- ☐ NORWEGIAN LUNDEHUND
- ☐ NORWEGIAN ELKHOUND, GREY
- ☐ NORWEGIAN ELKHOUND, BLACK
- ☐ NORWICH TERRIER
- ☐ NOVA SCOTIA DUCK TOLLING RETRIEVER
- ☐ OLD ENGLISH SHEEPDOG (BOBTAIL)
- ☐ OTTERHOUND
- ☐ PAPILLON
- ☐ PARSON RUSSELL TERRIER
- ☐ PEKINGESE
- ☐ PORTUGUESE POINTING DOG
- ☐ SPANISH WATERDOG
- ☐ MAJORCA MASTIFF
- ☐ PERUVIAN HAIRLESS DOG, LARGE
- ☐ PERUVIAN HAIRLESS DOG, MEDIUM-SIZE
- ☐ PERUVIAN HAIRLESS DOG, MINIATURE
- ☐ PETIT BASSET GRIFFON VENDÉEN
- ☐ SMALL BRABANT GRIFFON
- ☐ PHALÈNE
- ☐ GERMAN PINSCHER
- ☐ PLOTT
- ☐ IBIZAN HOUND, SMOOTH-HAIRED
- ☐ IBIZAN HOUND, ROUGH-HAIRED
- ☐ PORTUGUESE PODENGO (WARREN HOUND) WIRE-HAIRED/MEDIUM-SIZED
- ☐ PORTUGUESE PODENGO (WARREN HOUND) WIRE-HAIRED/MINIATURE
- ☐ PORTUGUESE PODENGO (WARREN HOUND) SMOOTH-HAIRED/MEDIUM-SIZED
- ☐ PORTUGUESE PODENGO (WARREN HOUND) SMOOTH-HAIRED/MINIATURE
- ☐ ENGLISH POINTER
- ☐ POLISH LOWLAND SHEEPDOG
- ☐ POMERANIAN
- ☐ PORCELAIN
- ☐ PORTUGUESE WATER DOG
- ☐ POSAVAZ HOUND
- ☐ PRAZSKÝ KRYSARÍK
- ☐ POODLE, MINIATURE
- ☐ POODLE, MEDIUM-SIZED
- ☐ POODLE, STANDARD
- ☐ POODLE, TOY

- ☐ PULI
- ☐ PUMI
- ☐ PYRENEAN MOUNTAIN DOG
- ☐ PYRENEAN MASTIFF
- ☐ RHODESIAN RIDGEBACK
- ☐ GIANT SCHNAUZER, PEPPER & SALT
- ☐ GIANT SCHNAUZER, BLACK
- ☐ ROTTWEILER
- ☐ RUSSIAN HOUND
- ☐ RUSSKAYA TSVETNAYA BOLONKA
- ☐ RUSSIAN TOY
- ☐ RYSK BLACK TERRIER
- ☐ RUSSIAN-EUROPEAN LAIKA
- ☐ SAARLOOS WOLFDOG
- ☐ SALUKI
- ☐ SAMOYED
- ☐ SAINT BERNARD DOG, SHORT-HAIRED
- ☐ SAINT BERNARD DOG, LONG-HAIRED
- ☐ SCHAPENDOES
- ☐ SCHILLER HOUND
- ☐ SCHILLER HOUND (RASVÅRD)
- ☐ SCHIPPERKE
- ☐ SCHNAUZER, PEPPER & SALT
- ☐ SCHNAUZER, BLACK
- ☐ SMALL SWISS HOUND/ LUCERNE HOUND
- ☐ SMALL SWISS HOUND/ SCHWYZ HOUND
- ☐ SEALYHAM TERRIER
- ☐ SHAR PEI
- ☐ SHETLAND SHEEPDOG
- ☐ SHIBA
- ☐ SHIH TZU
- ☐ SIBERIAN HUSKY
- ☐ AUSTRALIAN SILKY TERRIER
- ☐ SKOTTISH DEERHOUND
- ☐ SKOTTISH TERRIER
- ☐ SKYE TERRIER
- ☐ SLOUGHI
- ☐ SLOVAKIAN WIRE-HAIRED POINTING DOG
- ☐ SLOVAKIAN HOUND
- ☐ FOXTERRIER, SMOOTH
- ☐ SMÅLAND HOUND
- ☐ ITALIAN SPINONE
- ☐ CENTRAL ASIAN SHEPHERD DOG
- ☐ STABYHOUN
- ☐ STAFFORDSHIRE BULL TERRIER
- ☐ STYRIAN COARSE-HAIRED HOUND
- ☐ FOXTERRIER, WIRE
- ☐ GERMAN WIRE-HAIRED POINTER
- ☐ SUSSEX SPANIEL
- ☐ SWEDISH LAPPHUND
- ☐ SWEDISH WHITE ELKHOUND
- ☐ DACHSHUND
- ☐ DACHSHUND MINIATURE, SMOOTH-HAIRED
- ☐ DACHSHUND KANINCHEN, SMOOTH-HAIRED
- ☐ DACHSHUND STANDARD, SMOOTH-HAIRED
- ☐ DACHSHUND MINIATURE, LONG-HAIRED
- ☐ DACHSHUND KANINCHEN, LONG-HAIRED
- ☐ DACHSHUND STANDARD, LONG-HAIRED
- ☐ DACHSHUND MINIATURE, WIRE-HAIRED
- ☐ DACHSHUND KANINCHEN, WIRE-HAIRED
- ☐ DACHSHUND STANDARD, WIRE-HAIRED
- ☐ TENTERFIELD TERRIER
- ☐ BARZILIAN TERRIER
- ☐ THAI BANGKAEW DOG

- ☐ THAI RIDGEBACK DOG
- ☐ TIBETAN MASTIFF
- ☐ TIBETAN SPANIEL
- ☐ TIBETAN TERRIER
- ☐ TOSA
- ☐ GERMAN HUNTING TERRIER
- ☐ GERMAN SHEPHERD
- ☐ GERMAN SPITZ/ KLEIN
- ☐ GERMAN SPITZ/ MITTEL
- ☐ HUNGARIAN VIZSLA, SHORT-HAIRED
- ☐ HUNGARIAN VIZSLA, WIRE-HAIRED
- ☐ GERMAN SPANIEL
- ☐ WEIMARANER, SHORT-HAIRED
- ☐ WEIMARANER, LONG-HAIRED
- ☐ WELSH CORGI CARDIGAN
- ☐ WELSH CORGI PEMBROKE
- ☐ WELSH SPRINGER SPANIEL
- ☐ WELSH TERRIER
- ☐ WEST HIGHLAND WHITE TERRIER
- ☐ WHIPPET
- ☐ WHITE SWISS SHEPHERD DOG
- ☐ VOLPINO ITALIANO
- ☐ SWEDISH VALLHUND
- ☐ WEST SIBERIAN LAIKA
- ☐ MEXICAN HAIRLESS DOG, MINIATURE
- ☐ MEXICAN HAIRLESS DOG, INTERMEDIATE
- ☐ MEXICAN HAIRLESS DOG, STANDARD
- ☐ YORKSHIRE TERRIER
- ☐ AUSTRIAN PINSCHER
- ☐ EAST SIBERIAN LAIKA
- ☐ Other breeds

3. Approximately how much does your dog weigh?

- ☐ 0 - 2.9 kg
- ☐ 3 - 5.9 kg
- ☐ 6 - 9.9 kg
- ☐ 10 - 14.9 kg
- ☐ 15 - 19.9 kg
- ☐ 20 - 29.9 kg
- ☐ 30 kg or more
- ☐ Don´t know

4. What is your dog's gender?

- ☐ Female
- ☐ Male
- ☐ Female, neutered
- ☐ Male, neutered
- ☐ Don´t know

5. How would you appraise your dog's dental health ?

- ☐ Very bad
- ☐ Fairly bad
- ☐ Neither good nor bad
- ☐ Fairly good
- ☐ Very good
- ☐ **Don't know / Unable to judge**

6. How important is it for you that your dog has good dental health?

- ☐ Not at all important
- ☐ Of minor importance
- ☐ Fairly important
- ☐ Very important
- ☐ **Don't know**

7. What do you consider to be important for good dental health in dogs?

	Not at all important	Of minor importance	Fairly important	Very important	**Don't know**
**Good general health**	☐	☐	☐	☐	☐
**Special dog food that according to the manufacturer benefits dental health**	☐	☐	☐	☐	☐
**Chewingbones, e.g. rawhide, bully sticks, pig ears or pig tails**	☐	☐	☐	☐	☐
**Tooth brushing**	☐	☐	☐	☐	☐
**Dental cleaning with textiles (e.g. fingercloth, microfiber, cloth or gauze)**	☐	☐	☐	☐	☐
**The dogs breed / heredity**	☐	☐	☐	☐	☐
**Chewingtoys, e.g. squeaky toys, textile toys, artificial bones, stuffed toys**	☐	☐	☐	☐	☐
**Special dental chews that according to the manufacturer benefits dental health**	☐	☐	☐	☐	☐
**Marrow bones**	☐	☐	☐	☐	☐
**Dog toothpaste**	☐	☐	☐	☐	☐

8. Has it ever been recommended to you, by any of the following, to brush / clean your dog's teeth?

Brush refers to brush with toothbrush. Cleaning refers to cleaning with textiles, e.g. fingercloth, microfiber, other textiles or gauze.

	Yes	No	Don't know
**By a veterinary clinic**	☐	☐	☐
**By breeder**	☐	☐	☐
**By breed club/ dog club (e.g. member journal or lecture)**	☐	☐	☐
**By friends or family**	☐	☐	☐
**By books or journals**	☐	☐	☐
**By the internet (e.g. information pages or social media)**	☐	☐	☐
**By my own health care education**	☐	☐	☐

9. ***What method of dental cleaning was recommended at the veterinary clinic? (only visible to respondents who answered that they had been recommended to do so by a veterinary clinic on question 8)***

- ☐ Tootbrush
- ☐ Textiles (e.g. fingercloth, microfiber, cloth or gauze)
- ☐ Both toothbrush and textiles
- ☐ Don't know / Other

10. When you received the recommendation at the veterinary clinic to brush / clean your dog's teeth, who gave you the information? ***(only visible to respondents who answered that they had been recommended to do so by a veterinary clinic on question 8)***

- ☐ The veterinarian
- ☐ The veterinary nurse /animal care worker
- ☐ Both the veterinarian and the veterinary nurse / animal care worker
- ☐ Don't know / Other

11. When you received the recommendation at the veterinary clinic to brush / clean your dog's teeth, what was your primary reason for the visit? ***(only visible to respondents who answered that they had been recommended to do so by a veterinary clinic on question 8)***

Several options can be specified
- ☐ Puppy vaccination
- ☐ Other routine visit (e.g. vaccination))
- ☐ Visit for dental cleaning (calculus removal)/dental problems
- ☐ Visit due to other disease
- ☐ Special information meeting
- ☐ Don't know / Other

12. When you received the recommendation at the veterinary clinic to brush / clean your dog's teeth, how did you receive the information? ***(only visible to respondents who answered that they had been recommended to do so by a veterinary clinic on question 8)***

Several options can be specified
- ☐ Verbally
- ☐ Written
- ☐ Practical demonstration
- ☐ Information about web page or similar
- ☐ Don't know / Other

13. When you received the recommendation at the veterinary clinic: Did the information lead you to initiate brushing / cleaning your dog's teeth? ***(only visible to respondents who answered that they had been recommended to do so by a veterinary clinic on question 8)***

- ☐ I brushed / cleaned before I received the recommendation
- ☐ Yes, I brush / clean still
- ☐ Yes, I started (or tried) to brush / clean but stopped later
- ☐ No
- ☐ Don't know

14. How often in the last month have you brushed your dog's teeth with a toothbrush?

- ☐ Daily
- ☐ 4-6 days / week
- ☐ 1-3 days / week
- ☐ More seldom / single occasion
- ☐ Never
- ☐ Don't know

15. How often in the last month have you cleaned your dog's teeth with textiles (eg fingercloth, microfiber, other textiles or gauze)?

- Daily
- 4-6 days / week
- 1-3 days / week
- More seldom / single occasion
- Never
- Don't know

16. Would you consider brushing your dog's teeth daily? ***(not visible to respondents who answered "daily" on question 14)***

- Yes
- Maybe
- No
- Do not want to answer

17. Which of the following reasons for brushing the dog's teeth are the most important for you?

Enter the main reasons, max 3 options.

- To avoid anesthesia or surgery due to dental problems
- To avoid veterinary cost for dental problems
- That the dog should keep its teeth
- That the vet recommends it
- To avoid bad breath in the dog
- That it is good for the dog's general health
- **None of the above / Don't know**
- Other reason _________________________________

18. When you clean your dog's teeth at home, does the gum bleed? ***(not visible to respondents who answered "never" or "don´t know" on question 14 <u>and</u> 15)***

- No, never
- Yes, sometimes
- Yes, often
- Yes, always
- **Don't know / Do not want to answer**

19. How easy or difficult is it for you to inspect (look at) all of your dog's teeth?

- Very easy
- Fairly easy
- Fairly difficult
- Very difficult
- **Don't know**

20. What difficulties do you experience when inspecting your dog's teeth? (Only visible to respondents who answered "Fairly" or "Very difficult" on question 19)

Several options can be specified

- The dog is in pain
- The dog gets angry
- The dog doesn't want to
- Own impaired physical ability
- I do not know how to do it
- Technically / practically difficult to perform
- Don't know
- Other reason

21. How often in the last month has your dog chewed / used any of the following:

	Daily	4-6 days / week	1-3 days / week	More seldom / single occasion	Never	**Don't know**
**Chewingtoys, e.g. squeaky toys, textile toys, artificial bones, stuffed toys**	☐	☐	☐	☐	☐	☐
**Marrow bones**	☐	☐	☐	☐	☐	☐
**Chewingbones, e.g. rawhide, bully sticks, pig ears or pig tails**	☐	☐	☐	☐	☐	☐
**Special dental chews that according to the manufacturer benefits dental health**	☐	☐	☐	☐	☐	☐
**Dog toothpaste**	☐	☐	☐	☐	☐	☐
**Mouthwash or mouth gel with chlorhexidine**	☐	☐	☐	☐	☐	☐

22. Has a dental scaler been used by yourself or someone else (e.g. groomer or breeder) to remove the dog's calculus?
Note: This does not include calculus removal at a veterinary clinic.

☐ No
☐ Yes, once
☐ Yes, several times
☐ Don't know

23. Has your dog been anaesthetized at a veterinary clinic to clean the teeth / remove dental calculus?

☐ No
☐ Yes, once
☐ Yes, several times
☐ Don't know

24. Has your dog had problems with gum disease or loose teeth?
(Does not apply to puppy teeth)

☐ No
☐ Yes, the dog has had to extract teeth at the veterinary clinic and / or lost teeth at home
☐ Yes, but the dog has not had to extract any teeth
☐ Don't know

25. Does your dog have bad breath?

☐ No, never
☐ Yes, sometimes
☐ Yes, often
☐ Yes, always
☐ **Don't know**

26. Does your dog have dental calculus at the moment?

☐ No
☐ Yes, a little
☐ Yes, a moderate amount
☐ Yes, a lot
☐ **Don't know**

27. Have you been advised <u>at a veterinary clinic</u> to use any of the following to

improve your dog's dental health?

	Yes	No	**Don't know**
**Marrow bones**	☐	☐	☐
**Chewingbones, e.g. rawhide, bully sticks, pig ears or pig tails**	☐	☐	☐
**Special dental chews that according to the manufacturer benefits dental health**	☐	☐	☐
**Dog toothpaste**	☐	☐	☐
**Mouthwash or mouth gel with chlorhexidine**	☐	☐	☐
**Special dog food that according to the manufacturer benefits dental health**	☐	☐	☐

28. How would you appraise your dog's general health?

- ☐ Very bad
- ☐ Fairly bad
- ☐ Neither good nor bad
- ☐ Fairly good
- ☐ Very good
- ☐ **Don't know**

29. Does your dog have one or more of the following diseases:

Several options can be specified

- ☐ Diabetes (Diabetes mellitus)
- ☐ Cushing's disease
- ☐ Addison's disease
- ☐ Thyroid gland disease (Hypothyroidism)
- ☐ Heart disease
- ☐ Kidney disease
- ☐ Liver disease
- ☐ Skin disease, e.g. allergy
- ☐ Joint disease, e.g. arthrosis
- ☐ **None of the above mentioned diseases / Don't know**
- ☐ Other chronic disease ________________________________

30. Have you chosen to delay a visit to a veterinary clinic for dental problems, because of any of the following reasons?

Dental problems include calculus, gingivitis, gum disease, dental fractures and other dental diseases and injuries.

Several options can be specified

- ☐ Veterinary costs
- ☐ Did not have time
- ☐ Did not get an appointment immediately
- ☐ Negative experiences from previous visits
- ☐ Worry about the anesthesia / surgery
- ☐ Dog insurance doesn´t cover treatment
- ☐ Waited to see if the problem disappeared
- ☐ **No, did not delay / no need to visit a veterinarian because of dental problems**
- ☐ Don't know / Wishes not to answer / Other cause ________________________________

31. Are you a dog breeder?

- Yes
- No
- Don't know / Do not want to answer

32. Do you as a breeder recommend that puppy buyers brush their dog's teeth?
(Only visible to respondents who answered "Yes" on question 31)

- No, never
- Yes, sometimes
- Yes, often
- Yes, always
- **Don't know / Do not want to answer**

33. What year were you born?

Choose a year below

- 1926 or earlier
- 1927
- 1928
- 1929
- 1930
- 1931
- 1932
- 1933
- 1934
- 1935
- 1936
- 1937
- 1938
- 1939
- 1940
- 1941
- 1942
- 1943
- 1944
- 1945
- 1946
- 1947
- 1948
- 1949
- 1950
- 1951
- 1952
- 1953
- 1954
- 1955
- 1956
- 1957
- 1958
- 1959
- 1960
- 1961
- 1962
- 1963
- 1964
- 1965
- 1966
- 1967
- 1968
- 1969
- 1970
- 1971
- 1972
- 1973

- ☐ 1974
- ☐ 1975
- ☐ 1976
- ☐ 1977
- ☐ 1978
- ☐ 1979
- ☐ 1980
- ☐ 1981
- ☐ 1982
- ☐ 1983
- ☐ 1984
- ☐ 1985
- ☐ 1986
- ☐ 1987
- ☐ 1988
- ☐ 1989
- ☐ 1990
- ☐ 1991
- ☐ 1992
- ☐ 1993
- ☐ 1994
- ☐ 1995
- ☐ 1996
- ☐ 1997
- ☐ 1998
- ☐ 1999
- ☐ 2000 or later
- ☐ Do not want to answer

34. What is your gender?

- ☐ Female
- ☐ Male
- ☐ Do not want to answer / Other

35. What municipality do you live in?

Select municipality below

- ☐ Ale
- ☐ Alingsås
- ☐ Alvesta
- ☐ Aneby
- ☐ Arboga
- ☐ Arjeplog
- ☐ Arvidsjaur
- ☐ Arvika
- ☐ Askersund
- ☐ Avesta
- ☐ Bengtsfors
- ☐ Berg
- ☐ Bjurholm
- ☐ Bjuv
- ☐ Boden
- ☐ Bollebygd
- ☐ Bollnäs
- ☐ Borgholm
- ☐ Borlänge
- ☐ Borås
- ☐ Botkyrka
- ☐ Boxholm
- ☐ Bromölla
- ☐ Bräcke
- ☐ Burlöv
- ☐ Båstad

- [ ] Dals-Ed
- [ ] Danderyd
- [ ] Degerfors
- [ ] Dorotea
- [ ] Eda
- [ ] Ekerö
- [ ] Eksjö
- [ ] Emmaboda
- [ ] Enköping
- [ ] Eskilstuna
- [ ] Eslöv
- [ ] Essunga
- [ ] Fagersta
- [ ] Falkenberg
- [ ] Falköping
- [ ] Falun
- [ ] Filipstad
- [ ] Finspång
- [ ] Flen
- [ ] Forshaga
- [ ] Färgelanda
- [ ] Gagnef
- [ ] Gislaved
- [ ] Gnesta
- [ ] Gnosjö
- [ ] Gotland
- [ ] Grums
- [ ] Grästorp
- [ ] Gullspång
- [ ] Gällivare
- [ ] Gävle
- [ ] Göteborg
- [ ] Götene
- [ ] Habo
- [ ] Hagfors
- [ ] Hallsberg
- [ ] Hallstahammar
- [ ] Halmstad
- [ ] Hammarö
- [ ] Haninge
- [ ] Haparanda
- [ ] Heby
- [ ] Hedemora
- [ ] Helsingborg
- [ ] Herrljunga
- [ ] Hjo
- [ ] Hofors
- [ ] Huddinge
- [ ] Hudiksvall
- [ ] Hultsfred
- [ ] Hylte
- [ ] Håbo
- [ ] Hällefors
- [ ] Härjedalen
- [ ] Härnösand
- [ ] Härryda
- [ ] Hässleholm
- [ ] Höganäs
- [ ] Högsby
- [ ] Hörby
- [ ] Höör
- [ ] Jokkmokk
- [ ] Järfälla
- [ ] Jönköping

- [ ] Kalix
- [ ] Kalmar
- [ ] Karlsborg
- [ ] Karlshamn
- [ ] Karlskoga
- [ ] Karlskrona
- [ ] Karlstad
- [ ] Katrineholm
- [ ] Kil
- [ ] Kinda
- [ ] Kiruna
- [ ] Klippan
- [ ] Knivsta
- [ ] Kramfors
- [ ] Kristianstad
- [ ] Kristinehamn
- [ ] Krokom
- [ ] Kumla
- [ ] Kungsbacka
- [ ] Kungsör
- [ ] Kungälv
- [ ] Kävlinge
- [ ] Köping
- [ ] Laholm
- [ ] Landskrona
- [ ] Laxå
- [ ] Lekeberg
- [ ] Leksand
- [ ] Lerum
- [ ] Lessebo
- [ ] Lidingö
- [ ] Lidköping
- [ ] Lilla Edet
- [ ] Lindesberg
- [ ] Linköping
- [ ] Ljungby
- [ ] Ljusdal
- [ ] Ljusnarsberg
- [ ] Lomma
- [ ] Ludvika
- [ ] Luleå
- [ ] Lund
- [ ] Lycksele
- [ ] Lysekil
- [ ] Malmö
- [ ] Malung-Sälen
- [ ] Malå
- [ ] Mariestad
- [ ] Mark
- [ ] Markaryd
- [ ] Mellerud
- [ ] Mjölby
- [ ] Mora
- [ ] Motala
- [ ] Mullsjö
- [ ] Munkedal
- [ ] Munkfors
- [ ] Mölndal
- [ ] Mönsterås
- [ ] Mörbylånga
- [ ] Nacka
- [ ] Nora
- [ ] Norberg

- [ ] Nordanstig
- [ ] Nordmaling
- [ ] Norrköping
- [ ] Norrtälje
- [ ] Norsjö
- [ ] Nybro
- [ ] Nykvarn
- [ ] Nyköping
- [ ] Nynäshamn
- [ ] Nässjö
- [ ] Ockelbo
- [ ] Olofström
- [ ] Orsa
- [ ] Orust
- [ ] Osby
- [ ] Oskarshamn
- [ ] Ovanåker
- [ ] Oxelösund
- [ ] Pajala
- [ ] Partille
- [ ] Perstorp
- [ ] Piteå
- [ ] Ragunda
- [ ] Robertsfors
- [ ] Ronneby
- [ ] Rättvik
- [ ] Sala
- [ ] Salem
- [ ] Sandviken
- [ ] Sigtuna
- [ ] Simrishamn
- [ ] Sjöbo
- [ ] Skara
- [ ] Skellefteå
- [ ] Skinnskatteberg
- [ ] Skurup
- [ ] Skövde
- [ ] Smedjebacken
- [ ] Sollefteå
- [ ] Sollentuna
- [ ] Solna
- [ ] Sorsele
- [ ] Sotenäs
- [ ] Staffanstorp
- [ ] Stenungsund
- [ ] Stockholm
- [ ] Storfors
- [ ] Storuman
- [ ] Strängnäs
- [ ] Strömstad
- [ ] Strömsund
- [ ] Sundbyberg
- [ ] Sundsvall
- [ ] Sunne
- [ ] Surahammar
- [ ] Svalöv
- [ ] Svedala
- [ ] Svenljunga
- [ ] Säffle
- [ ] Säter
- [ ] Sävsjö
- [ ] Söderhamn
- [ ] Söderköping
- [ ] Södertälje

- [ ] Sölvesborg
- [ ] Tanum
- [ ] Tibro
- [ ] Tidaholm
- [ ] Tierp
- [ ] Timrå
- [ ] Tingsryd
- [ ] Tjörn
- [ ] Tomelilla
- [ ] Torsby
- [ ] Torsås
- [ ] Tranemo
- [ ] Tranås
- [ ] Trelleborg
- [ ] Trollhättan
- [ ] Trosa
- [ ] Tyresö
- [ ] Täby
- [ ] Töreboda
- [ ] Uddevalla
- [ ] Ulricehamn
- [ ] Umeå
- [ ] Upplands Väsby
- [ ] Upplands-Bro
- [ ] Uppsala
- [ ] Uppvidinge
- [ ] Vadstena
- [ ] Vaggeryd
- [ ] Valdemarsvik
- [ ] Vallentuna
- [ ] Vansbro
- [ ] Vara
- [ ] Varberg
- [ ] Vaxholm
- [ ] Vellinge
- [ ] Vetlanda
- [ ] Vilhelmina
- [ ] Vimmerby
- [ ] Vindeln
- [ ] Vingåker
- [ ] Vårgårda
- [ ] Vänersborg
- [ ] Vännäs
- [ ] Värmdö
- [ ] Värnamo
- [ ] Västervik
- [ ] Västerås
- [ ] Växjö
- [ ] Ydre
- [ ] Ystad
- [ ] Åmål
- [ ] Ånge
- [ ] Åre
- [ ] Årjäng
- [ ] Åsele
- [ ] Åstorp
- [ ] Åtvidaberg
- [ ] Älmhult
- [ ] Älvdalen
- [ ] Älvkarleby
- [ ] Älvsbyn
- [ ] Ängelholm
- [ ] Öckerö

- ☐ Ödeshög
- ☐ Örebro
- ☐ Örkelljunga
- ☐ Örnsköldsvik
- ☐ Östersund
- ☐ Österåker
- ☐ Östhammar
- ☐ Östra Göinge
- ☐ Överkalix
- ☐ Övertorneå
- ☐ Don't know

36. What is your main employment?

- ☐ Student
- ☐ Part-time employee
- ☐ Full-time employee
- ☐ Self-employed
- ☐ Permanent or parental leave
- ☐ Long-term sick leave (more than 3 months) or sick-pensioner
- ☐ Unemployed
- ☐ Retired
- ☐ Don't know / Do not want to answer
- ☐ Other employment

37. What is your highest level of education?

- ☐ Not completed primary school
- ☐ Elementary school or equivalent
- ☐ Gymnasium
- ☐ Vocational training
- ☐ University or college less than 3 years
- ☐ University or college 3 years or more
- ☐ Don't know / Do not want to answer

38. Are you a qualified practitioner in any of the following healthcare professions?

Several options can be specified

- ☐ **No**
- ☐ Assistant nurse
- ☐ Nurse
- ☐ Physician
- ☐ Dental nurse
- ☐ Dental hygienist
- ☐ Dentist
- ☐ Animal carer
- ☐ Veterinary nurse
- ☐ Veterinarian
- ☐ Other health care profession ________________________________

39. If you have any further information you would like to share, please feel free to write this below.

____
____
____
____
____

Sveriges lantbruksuniversitet
Swedish University of Agricultural Sciences

Thank you for your participation!

Your answers will contribute to the research and increase our knowledge of dental health in dogs.

Kind regards,

Ann Pettersson, head researcher
Associate Professor, DVM, PhD, Swedish Specialist odontology dogs and cats

Karolina Enlund
PhD student, DVM

Sveriges lantbruksuniversitet
Swedish University of Agricultural Sciences
Department of clinical sciences
Box 7054, 750 07 UPPSALA
Visiting address: Ulls väg 26

Tel: 018-67 10 00
tandhalsahoshund@slu.se
www.slu.se

Click on the link to get to the project website:

http://www.slu.se/fakulteter/vh/forskning/forskningsprojekt/hund/Tandhalsa-hos-hund/

Tandhälsa hos hund

Sveriges lantbruksuniversitet
Swedish University of Agricultural Sciences

Welcome to our survey about canine dental health!

Welcome to the canine dental health survey! This survey is sent to you in your role as a veterinarian or veterinary nurse, irrespective of your current occupation. We were passed your details by the Swedish Board of Agriculture. The survey should take about 5-10 minutes to complete and the answers are only used at a group level. Your answers are vital for our research on how veterinarians / veterinary nurses experience dental health in dogs. If you choose not to take part we cannot replace you with any other respondents, so we would be extremely grateful for your participation.

Sincerely,

Ann Pettersson, head researcher
Associate Professor, DVM, PhD, Swedish Specialist odontology dogs and cats

Karolina Enlund
PhD student, DVM

Sveriges lantbruksuniversitet
Swedish University of Agricultural Sciences
Department of clinical sciences
Box 7054, 750 07 UPPSALA
Visiting address: Ulls väg 26

Tel: 018-67 10 00
tandhalsahoshund@slu.se
www.slu.se

Research Institution: Swedish University of Agricultural Sciences. Participation is voluntary and can be terminated at any time without any consequences. No unauthorized person will know how you answered.
The Swedish University of Agricultural Sciences is responsible for personal data relating to this survey. The Personal Data Act, PUL (1998: 204), states that once ayear you are entitled to receive, free of charge, details of all the information about yourself that is beeing processed and, if necessary, have any errors corrected. Contact person: Ann Pettersson, ann.pettersson@slu.se, 018-67 10 00. The project is funded by Stiftelsen Djursjukhus i Stor Stockholm and Svenska Djurskyddsföreningen.

1. Year of birth

Choose year below

- ☐ 1937 or earlier
- ☐ 1938
- ☐ 1939
- ☐ 1940
- ☐ 1941
- ☐ 1942
- ☐ 1943
- ☐ 1944
- ☐ 1945
- ☐ 1946
- ☐ 1947
- ☐ 1948
- ☐ 1949
- ☐ 1950
- ☐ 1951
- ☐ 1952
- ☐ 1953
- ☐ 1954
- ☐ 1955
- ☐ 1956
- ☐ 1957
- ☐ 1958
- ☐ 1959
- ☐ 1960
- ☐ 1961
- ☐ 1962
- ☐ 1963
- ☐ 1964
- ☐ 1965
- ☐ 1966
- ☐ 1967
- ☐ 1968
- ☐ 1969
- ☐ 1970
- ☐ 1971
- ☐ 1972
- ☐ 1973
- ☐ 1974
- ☐ 1975
- ☐ 1976
- ☐ 1977
- ☐ 1978
- ☐ 1979
- ☐ 1980
- ☐ 1981
- ☐ 1982
- ☐ 1983
- ☐ 1984
- ☐ 1985
- ☐ 1986
- ☐ 1987
- ☐ 1988
- ☐ 1989
- ☐ 1990
- ☐ 1991
- ☐ 1992
- ☐ 1993
- ☐ Prefer not to answer

2. When did you receive your degree in veterinary medicine / nursing?

Choose year below

- ☐ 1975 or earlier
- ☐ 1976
- ☐ 1977
- ☐ 1978
- ☐ 1979
- ☐ 1980
- ☐ 1981
- ☐ 1982
- ☐ 1983
- ☐ 1984
- ☐ 1985
- ☐ 1986
- ☐ 1987
- ☐ 1988
- ☐ 1989
- ☐ 1990
- ☐ 1991
- ☐ 1992
- ☐ 1993
- ☐ 1994
- ☐ 1995
- ☐ 1996
- ☐ 1997
- ☐ 1998
- ☐ 1999
- ☐ 2000
- ☐ 2001
- ☐ 2002
- ☐ 2003
- ☐ 2004
- ☐ 2005
- ☐ 2006
- ☐ 2007
- ☐ 2008
- ☐ 2009
- ☐ 2010
- ☐ 2011
- ☐ 2012
- ☐ 2013
- ☐ 2014
- ☐ 2015
- ☐ 2016
- ☐ 2017
- ☐ Prefer not to answer

3. What is your gender?

- ☐ Female
- ☐ Male
- ☐ Prefer not to answer / Other

4. Which municipality do you live in?

Choose municipality below

- ☐ Ale
- ☐ Alingsås
- ☐ Alvesta
- ☐ Aneby
- ☐ Arboga
- ☐ Arjeplog
- ☐ Arvidsjaur
- ☐ Arvika
- ☐ Askersund

- [ ] Avesta
- [ ] Bengtsfors
- [ ] Berg
- [ ] Bjurholm
- [ ] Bjuv
- [ ] Boden
- [ ] Bollebygd
- [ ] Bollnäs
- [ ] Borgholm
- [ ] Borlänge
- [ ] Borås
- [ ] Botkyrka
- [ ] Boxholm
- [ ] Bromölla
- [ ] Bräcke
- [ ] Burlöv
- [ ] Båstad
- [ ] Dals-Ed
- [ ] Danderyd
- [ ] Degerfors
- [ ] Dorotea
- [ ] Eda
- [ ] Ekerö
- [ ] Eksjö
- [ ] Emmaboda
- [ ] Enköping
- [ ] Eskilstuna
- [ ] Eslöv
- [ ] Essunga
- [ ] Fagersta
- [ ] Falkenberg
- [ ] Falköping
- [ ] Falun
- [ ] Filipstad
- [ ] Finspång
- [ ] Flen
- [ ] Forshaga
- [ ] Färgelanda
- [ ] Gagnef
- [ ] Gislaved
- [ ] Gnesta
- [ ] Gnosjö
- [ ] Gotland
- [ ] Grums
- [ ] Grästorp
- [ ] Gullspång
- [ ] Gällivare
- [ ] Gävle
- [ ] Göteborg
- [ ] Götene
- [ ] Habo
- [ ] Hagfors
- [ ] Hallsberg
- [ ] Hallstahammar
- [ ] Halmstad
- [ ] Hammarö
- [ ] Haninge
- [ ] Haparanda
- [ ] Heby
- [ ] Hedemora
- [ ] Helsingborg
- [ ] Herrljunga
- [ ] Hjo
- [ ] Hofors

- [ ] Huddinge
- [ ] Hudiksvall
- [ ] Hultsfred
- [ ] Hylte
- [ ] Håbo
- [ ] Hällefors
- [ ] Härjedalen
- [ ] Härnösand
- [ ] Härryda
- [ ] Hässleholm
- [ ] Höganäs
- [ ] Högsby
- [ ] Hörby
- [ ] Höör
- [ ] Jokkmokk
- [ ] Järfälla
- [ ] Jönköping
- [ ] Kalix
- [ ] Kalmar
- [ ] Karlsborg
- [ ] Karlshamn
- [ ] Karlskoga
- [ ] Karlskrona
- [ ] Karlstad
- [ ] Katrineholm
- [ ] Kil
- [ ] Kinda
- [ ] Kiruna
- [ ] Klippan
- [ ] Knivsta
- [ ] Kramfors
- [ ] Kristianstad
- [ ] Kristinehamn
- [ ] Krokom
- [ ] Kumla
- [ ] Kungsbacka
- [ ] Kungsör
- [ ] Kungälv
- [ ] Kävlinge
- [ ] Köping
- [ ] Laholm
- [ ] Landskrona
- [ ] Laxå
- [ ] Lekeberg
- [ ] Leksand
- [ ] Lerum
- [ ] Lessebo
- [ ] Lidingö
- [ ] Lidköping
- [ ] Lilla Edet
- [ ] Lindesberg
- [ ] Linköping
- [ ] Ljungby
- [ ] Ljusdal
- [ ] Ljusnarsberg
- [ ] Lomma
- [ ] Ludvika
- [ ] Luleå
- [ ] Lund
- [ ] Lycksele
- [ ] Lysekil
- [ ] Malmö
- [ ] Malung-Sälen

- [ ] Malå
- [ ] Mariestad
- [ ] Mark
- [ ] Markaryd
- [ ] Mellerud
- [ ] Mjölby
- [ ] Mora
- [ ] Motala
- [ ] Mullsjö
- [ ] Munkedal
- [ ] Munkfors
- [ ] Mölndal
- [ ] Mönsterås
- [ ] Mörbylånga
- [ ] Nacka
- [ ] Nora
- [ ] Norberg
- [ ] Nordanstig
- [ ] Nordmaling
- [ ] Norrköping
- [ ] Norrtälje
- [ ] Norsjö
- [ ] Nybro
- [ ] Nykvarn
- [ ] Nyköping
- [ ] Nynäshamn
- [ ] Nässjö
- [ ] Ockelbo
- [ ] Olofström
- [ ] Orsa
- [ ] Orust
- [ ] Osby
- [ ] Oskarshamn
- [ ] Ovanåker
- [ ] Oxelösund
- [ ] Pajala
- [ ] Partille
- [ ] Perstorp
- [ ] Piteå
- [ ] Ragunda
- [ ] Robertsfors
- [ ] Ronneby
- [ ] Rättvik
- [ ] Sala
- [ ] Salem
- [ ] Sandviken
- [ ] Sigtuna
- [ ] Simrishamn
- [ ] Sjöbo
- [ ] Skara
- [ ] Skellefteå
- [ ] Skinnskatteberg
- [ ] Skurup
- [ ] Skövde
- [ ] Smedjebacken
- [ ] Sollefteå
- [ ] Sollentuna
- [ ] Solna
- [ ] Sorsele
- [ ] Sotenäs
- [ ] Staffanstorp
- [ ] Stenungsund
- [ ] Stockholm
- [ ] Storfors

- ☐ Storuman
- ☐ Strängnäs
- ☐ Strömstad
- ☐ Strömsund
- ☐ Sundbyberg
- ☐ Sundsvall
- ☐ Sunne
- ☐ Surahammar
- ☐ Svalöv
- ☐ Svedala
- ☐ Svenljunga
- ☐ Säffle
- ☐ Säter
- ☐ Sävsjö
- ☐ Söderhamn
- ☐ Söderköping
- ☐ Södertälje
- ☐ Sölvesborg
- ☐ Tanum
- ☐ Tibro
- ☐ Tidaholm
- ☐ Tierp
- ☐ Timrå
- ☐ Tingsryd
- ☐ Tjörn
- ☐ Tomelilla
- ☐ Torsby
- ☐ Torsås
- ☐ Tranemo
- ☐ Tranås
- ☐ Trelleborg
- ☐ Trollhättan
- ☐ Trosa
- ☐ Tyresö
- ☐ Täby
- ☐ Töreboda
- ☐ Uddevalla
- ☐ Ulricehamn
- ☐ Umeå
- ☐ Upplands Väsby
- ☐ Upplands-Bro
- ☐ Uppsala
- ☐ Uppvidinge
- ☐ Vadstena
- ☐ Vaggeryd
- ☐ Valdemarsvik
- ☐ Vallentuna
- ☐ Vansbro
- ☐ Vara
- ☐ Varberg
- ☐ Vaxholm
- ☐ Vellinge
- ☐ Vetlanda
- ☐ Vilhelmina
- ☐ Vimmerby
- ☐ Vindeln
- ☐ Vingåker
- ☐ Vårgårda
- ☐ Vänersborg
- ☐ Vännäs
- ☐ Värmdö
- ☐ Värnamo
- ☐ Västervik

- Västerås
- Växjö
- Ydre
- Ystad
- Åmål
- Ånge
- Åre
- Årjäng
- Åsele
- Åstorp
- Åtvidaberg
- Älmhult
- Älvdalen
- Älvkarleby
- Älvsbyn
- Ängelholm
- Öckerö
- Ödeshög
- Örebro
- Örkelljunga
- Örnsköldsvik
- Östersund
- Österåker
- Östhammar
- Östra Göinge
- Överkalix
- Övertorneå
- Don't know

5. Do you meet dogs in your professional role as a veterinarian / veterinary nurse?

- No, never
- Yes, sometimes
- Yes, often
- **Don't know / Can't answer question**

6. Do you work (regardless of how much) in a pet clinic or animal hospital for dogs, cats and smaller animals? ***(only visible to respondents who answered yes on question 5)***

- Yes
- No
- **Don´t know / Prefer not to answer**

7. What is the approximate size of the pet clinic/animal hospital where you work? ***(only visible to respondents who answered yes on question 6)***

Corresponding to full time positions

- 1 veterinarian
- 2 veterinarians
- 3-5 veterinarians
- 6-10 veterinarians
- 11-30 veterinarians
- 31 veterinarians or more
- **Don't know / Other**

8. What do you consider to be important for good dental health in dogs? ***(visible to all respondents, rest of questionnaire only visible to respondents who answered yes on question 5)***

	Not at all important	Not very important	Sometimes important	Fairly important	Very important	**Don't know**
**Good general health**	☐	☐	☐	☐	☐	☐
**Special dog food that according to the manufacturer benefits dental health**	☐	☐	☐	☐	☐	☐
**Chewing bones, e.g. rawhide, bully sticks, pig ears or pig tails.**	☐	☐	☐	☐	☐	☐
**Tooth brushing**	☐	☐	☐	☐	☐	☐
**Dental cleaning with textiles (e.g. fingercloth, microfiber, cloth or gauze)**	☐	☐	☐	☐	☐	☐
**Dog breed / heredity**	☐	☐	☐	☐	☐	☐
**Chewing toys, e.g. squeaky toys, textile toys, artificial bones, stuffed toys**	☐	☐	☐	☐	☐	☐
**Special dental chews that according to the manufacturer benefits dental health**	☐	☐	☐	☐	☐	☐
**Marrow bones**	☐	☐	☐	☐	☐	☐
**Dog toothpaste**	☐	☐	☐	☐	☐	☐
**Mouthwash or mouth gel containing chlorhexidine**	☐	☐	☐	☐	☐	☐
**Regular dental cleaning under anesthesia at a veterinary clinic**	☐	☐	☐	☐	☐	☐

9. How common or uncommon do you consider dental problems to be in the dogs you meet?

Problems include calculus, gingivitis, gum disease, tooth fractures and other dental diseases and injuries.

☐ Very uncommon
☐ Fairly uncommon
☐ Fairly common
☐ Very common
☐ **Don't know**

10. How common or uncommon do you consider gum disease (periodontal disease) to be in the dogs you meet?

- ☐ Very uncommon
- ☐ Fairly uncommon
- ☐ Fairly common
- ☐ Very common
- ☐ **Don't know**

11. Do you recommend that dog owners use any of the following to improve the dog's dental health?

	No, never	Yes, sometimes	Yes, often	Yes, always	**Don't know**
**Special dog food that according to the manufacturer benefits dental health**	☐	☐	☐	☐	☐
**Chewingbones, e.g. rawhide, bully sticks, pig ears or pig tails**	☐	☐	☐	☐	☐
**Tooth brushing**	☐	☐	☐	☐	☐
**Dental cleaning with textiles, e.g. fingercloth, microfiber, cloth or gauze**	☐	☐	☐	☐	☐
**Special dental chews that according to the manufacturer benefits dental health**	☐	☐	☐	☐	☐
**Marrow bones**	☐	☐	☐	☐	☐
**Dog toothpaste**	☐	☐	☐	☐	☐
**Mouthwash or mouth gel with chlorhexidine**	☐	☐	☐	☐	☐

12. At your workplace: Do veterinarians *(in the nurse survey)*/ veterinary nurses *(in the vet survey)* recommend that dog owners clean their dog's teeth with the following :

	No, never	Yes, sometimes	Yes, often	Yes, always	**Don't know/ No such staff**
**Toothbrush**	☐	☐	☐	☐	☐
**Dental cleaning with textiles, e.g. fingercloth, microfiber, cloth or gauze**	☐	☐	☐	☐	☐

13. Which of the following methods do you primarily recommend?

- ☐ Toothbrush
- ☐ Textiles (e.g. fingercloth, microfiber, cloth or gauze)
- ☐ Both toothbrush and textiles
- ☐ Don´t know / Other

14. When do you provide information about dental cleaning (with toothbrush or textiles) to dog owners?

Several options can be specified

- ☐ Puppy vaccination
- ☐ Other routine visit (e.g. vaccination))
- ☐ Visit for dental cleaning (calculus removal)
- ☐ Visit due to dental problems
- ☐ Visit due to other disease
- ☐ Special information meeting
- ☐ Don't know / Other

15. <u>How</u> do you provide information about dental cleaning (with toothbrush or textiles) to dog owners?

Several options can be specified

- ☐ Verbally
- ☐ Written
- ☐ Practical demonstration
- ☐ Information about web pages or similar
- ☐ Don't know / Do not want to answer
- ☐ Other ________________________________

16. What dental cleaning frequency do you recommend?

- ☐ Daily
- ☐ Every other day
- ☐ Once a week
- ☐ As often as they have time for
- ☐ I don't specify
- ☐ Don't know / Do not want to answer
- ☐ Annat ________________________________

17. Do you follow up whether the dog owner is satisfactorily performing home dental care on the dog?

Follow up means checking if the dog owner is carrying out dental home care on the dog, eg, via telephone call, email, visit or re-visit.

- ☐ No, never
- ☐ Yes, sometimes
- ☐ Yes, often
- ☐ Yes, always
- ☐ **Don't know / Do not want to answer**

18. Is dental cleaning performed with ultrasonic scaler on dogs under <u>sedation</u> (e.g. dexmedetomidine / butorphanol) done at your workplace?

Several options are possible

- ☐ Yes, by me
- ☐ Yes, by another veterinary nurse *(in the nurse survey)* /veterinarian *(in the vet survey)*
- ☐ Yes, by the veterinarian *(in the nurse survey)* /veterinary nurse *(in the vet survey)*
- ☐ No
- ☐ Don´t know / Do not want to answer

19. Is dental cleaning performed with ultrasonic scaler on dogs <u>under general anaesthesia</u> at your workplace?

Several options can be specified

- ☐ Yes, by me
- ☐ Yes, by another veterinary nurse *(in the nurse survey)* /veterinarian *(in the vet survey)*
- ☐ Yes, by the veterinarian *(in the nurse survey)* /veterinary nurse *(in the vet survey)*
- ☐ No
- ☐ Don´t know / Do not want to answer

20. What type of general anesthesia is used when performing dental cleaning with ultrasonic scaler? *(Question visible only if question 19 is answered yes)*

Several options can be specified

- ☐ Inhalation anesthetic (gas)
- ☐ Dissociative anesthetic (ketamin)
- ☐ Total Intravenous Anesthesia (TIVA, e.g. propofol)
- ☐ Don't know / Do not want to answer

21. At your workplace: Are teeth sometimes extracted when performing a tooth cleaning?

Several options can be specified

- ☐ Yes, by me
- ☐ Yes, by another veterinary nurse *(in the vet nurse survey)* /veterinarian *(in the vet survey)*
- ☐ Yes, by the veterinarian *(in the vet nurse survey)* /veterinary nurse *(in the vet survey)*
- ☐ No
- ☐ Don't know / Do not want to answer

22. Do you have access to a dental x-ray unit?

- ☐ Yes
- ☐ No
- ☐ Don´t know / Do not want to answer

23. What do you consider to be the most common reason for dog owners <u>not</u> to brush their dog's teeth?

- ☐ Dog unwilling / difficult to handle
- ☐ Ignorance
- ☐ Lack of time
- ☐ Dog owner considers toothbrushing unimportant
- ☐ Don't know / Do not want to answer
- ☐ Other ________________________________

24. What do you consider the most common reasons why veterinary nurses *(to vet nurses)* / veterinarians *(to vets)* do <u>not</u> inform dog owners about tooth brushing?

Maximum 3 options can be specified

- ☐ Lack of time
- ☐ Considered unimportant
- ☐ Dog owner could take offense
- ☐ Dog behaviour expected to make toothbrushing impossible
- ☐ Dog owner not expected to manage toothbrushing on dog
- ☐ Occasion not suitable
- ☐ Forgets
- ☐ Don´t know / Do not want to answer
- ☐ Other reason ________________________________

25. If you have any further information you would like to share, please feel free to write this below.

____
____
____
____
____

SLU Sveriges lantbruksuniversitet
Swedish University of Agricultural Sciences

Thank you for your participation!

Your answers will contribute to the research and increase our knowledge of dental health in dogs.

Kind regards,

Ann Pettersson, head researcher
Associate Professor, DVM, PhD, Swedish Specialist odontology dogs and cats

Karolina Enlund
PhD student, DVM

Sveriges lantbruksuniversitet
Swedish University of Agricultural Sciences
Department of clinical sciences
Box 7054, 750 07 UPPSALA
Visiting address: Ulls väg 26

Tel: 018-67 10 00
tandhalsahoshund@slu.se
www.slu.se

Click on the link to get to the project website:

http://www.slu.se/fakulteter/vh/forskning/forskningsprojekt/hund/Tandhalsa-hos-hund/
