# Supplementary material for: Development and validation of two questionnaires: Dental home care and dental health in Swedish dogs
Source: PLoS One. 2019 Jan 25;14(1):e0204581. doi: 10.1371/journal.pone.0204581 (PMC6347148; doi:10.1371/journal.pone.0204581)
Supplement: S2 Doc — (DOCX) [file pone.0204581.s002.docx]

# Survey Construction - Detailed

## Background

To determine the validity of acquired results in a survey, both *sampling error*, *coverage error*, *measurement error*s and *non-response error* must be evaluated (1). If rigorous methodology is not applied, the risk of obtaining low quality data, e.g. due to low representability, social desirability and other biases, is imminent. Furthermore, researchers’ desire to collect high quality and high-resolution data needs to be balanced against respondent burden, which should be kept to a minimum to ensure a high completion rate (2).

In order to avoid *sampling* and *coverage error* and for conclusions to be valid, the sample frame (all those within a population who can be sampled) should be representative of the target population and the respondents (the completed sample) should be representative of the sample frame and target population (1). A total sample, i.e. where the sample frame is equal to the total population, is generally not necessary in order to achieve representativity. The number of respondents needed to obtain significant results depends on problem definition and is not always possible to predict if the survey topic is new. A larger sample is often, but not always, more reliable (2). The actual sample frame is affected by both over- and under-coverage, i.e. some of the recipients of a questionnaire may not belong in the target group and some potential respondents on the recipient list may not receive a questionnaire, for example due to technical error (1). In conclusion, the statistical power of the study is largely dependent on the number of respondents but also the statistical precision, i.e. the representativity (2).

*Measurement errors* occur when the collected data contains inaccurate or incomplete information, due to e.g. poor question wording or imperfect response scales (3).

With higher response rates, the risk of *non-response error* is lower. Response rates are also essential for ensuring the validity of the results, and are affected by both external (not answering the questionnaire at all) and internal (incompletely answered questionnaire) non-response. Internal non-response can be random or systematic, of which the latter poses more severe problems when analysing results. Several studies have identified effective strategies to minimise non-response and thus increase the response rate of a questionnaire survey (4). Discrepancies between the response group and the total population are common, and therefore a non-response analysis is recommended in order to determine the representativity of the respondents. The non-response analysis should consist of, at least, a comparison between the response group and the sample frame, or target population, regarding e.g. age and gender (2).

The overall consistency of a questionnaire, i.e. the *reliability* (Fig. 1), is commonly assessed by test-retest, which assesses the stability of item responses over time and thus provides information on repeatability of the study. A questionnaire item may here be defined as an individual question with associated response options. Reliability can also be measured by inter-rater agreement, meaning the consistency between different assessors in assessed responses. Normally, several items are combined to represent a so-called construct, in which an underlying idea or theory containing various conceptual elements is assembled. The internal consistency of a construct, i.e. to what extent the selected items measure a single latent construct, is generally measured by Cronbach’s alpha (2) which ranges from 0 (complete lack of internal consistency) to 1 (absolute internal consistency). Although Cronbach’s alpha has received criticism as a measure of internal consistency (5), it is still a widely used metric.

**Fig. 1. Model of questionnaire evaluation: Different concepts of validity and reliability of a questionnaire.**

**
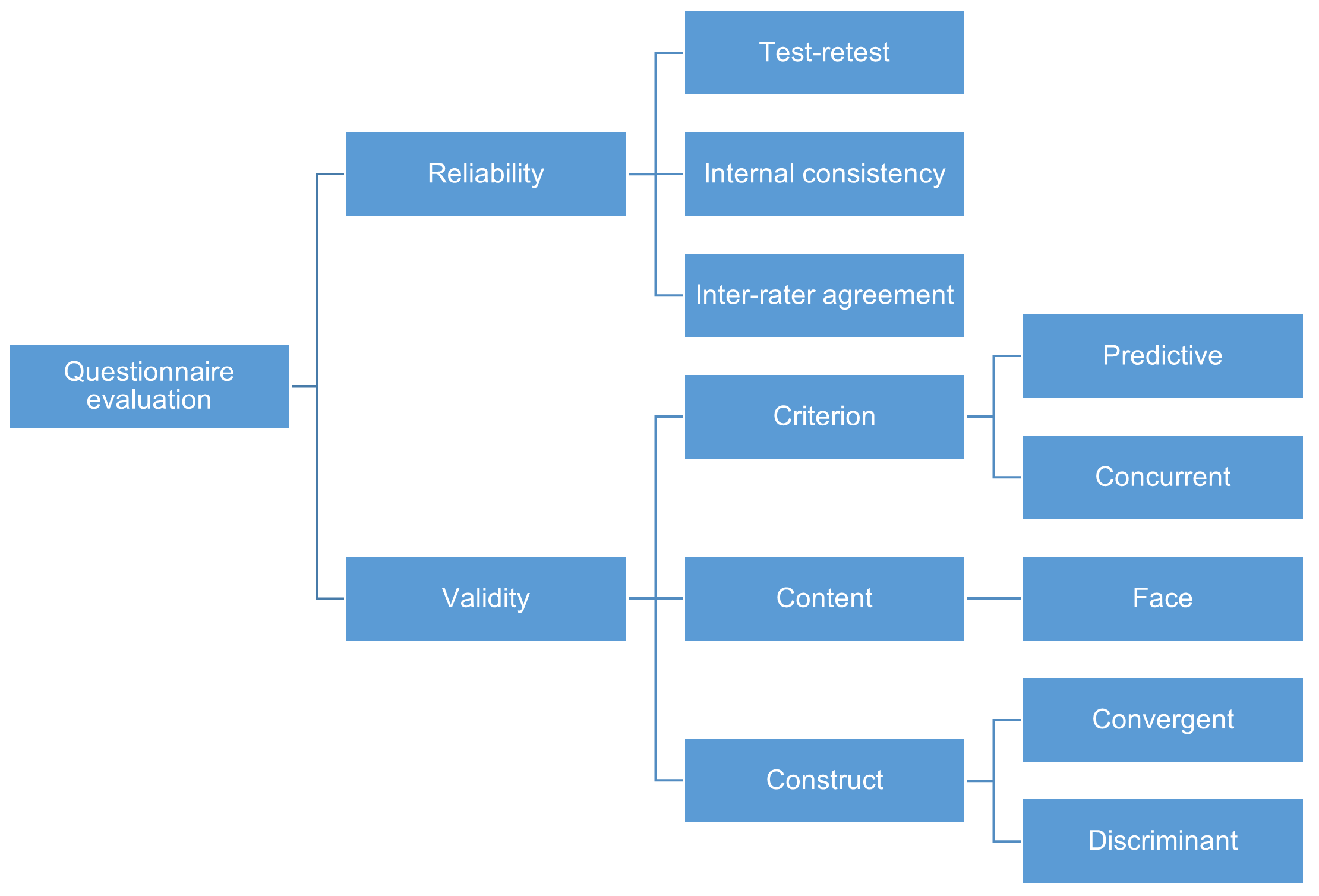
**

*Content, construct* and *criterion validity* (Fig. 1) are used to describe whether a questionnaire actually measures what it is intended to as seen from different aspects. The *content validity*, i.e. that a test measures all facets of a given construct, is usually assessed by Subject-Matter Experts (SME). This approach is often combined with cognitive interviews with representatives of the target population, e.g. using “think-aloud” (2, 6) or other focus group methods (6). Cognitive interviews may also be used to ensure face validity, i.e. that the test subjectively covers the intended concept. *Construct validity* can be described as the extent to which the theoretical knowledge basis is in fact operationalised, whereas *criterion validity* pertains to the relation of the measure to a pre-specified outcome (2, 7).

## Methods

### Number and order of items

The questionnaire was shorter (17 questions) or longer (up to 68 questions) for individual respondents depending on their previous answers (Supporting information). For instance, veterinarians with no dog practice were only asked background and attitude questions. The questionnaires were constructed so as not to require more than approximately 15 minutes’ completion time.

In the dog owner questionnaire, background questions concerning the dog that were deemed to be easily answered (such as age, breed and sex), were placed at the beginning, followed by general questions which focused on the study’s aims (such as assessment of dental health in the dog), then attitudes to dental home care and later practices of dental care. Socio-demographic characteristics of the respondent were placed last. In the questionnaire to veterinarians and veterinary nurses, the background questions (such as age and gender) were placed first. The order of the remaining questions was similar in both surveys (Supporting information).

### Construction of questions and response options

When formulating survey items, efforts were made to avoid provoking negative emotional responses in relation to personal questions, e.g. by asking for year of birth instead of age, and adapting gender options from recommendations by the Swedish Federation for Lesbian, Gay, Bisexual and Transgender Rights (8). Questions concerning education, occupation and municipality were adapted from a Netigate template and Statistics Sweden (SCB) / The Public Health Agency of Sweden (Folkhälsomyndigheten) surveys.

In order to minimise measurement errors, the following basic guidelines were applied as far as possible: Efforts were made to ensure that the order of the questions followed a logical sequence. Negations, difficult words, abbreviations and technical terms were avoided as far as possible and technical terms were defined and explained if they could not be avoided. Neutral questions were used to avoid acquiescence bias (“yea-saying”). Questions were specific and reference dates / periods, when applicable, clearly stated and kept unchanged (1 month) (Supporting information). Positions of answer boxes and direction within response alternatives were unaltered between questions, for example going from seldom (left) to often (right) or bad (left) to good (right), and the same sort and number of response alternatives were reused as far as possible. Care was taken to use clear and simple language, with short questions, formulated so that respondents would be able to give a clear answer. The response options were formulated to cover all conceivable options and to be mutually exclusive. A “don’t know” alternative was included as an option when applicable (8). Summarising questions (i.e. asking two questions in one) and abstractions were avoided. Although potentially difficult to interpret, hypothetical questions were used in specific cases, such as “Would you be willing to brush your dog’s teeth?”

Because of the diversity in data collected, both nominal-, ordinal- and interval-scales were used as response options (Supporting information). Where the response alternatives were fixed statements, e.g. when asking for sources of information on dog tooth brushing, the order of the options was randomised by the web platform to each respondent in order to avoid answer order bias (priming). Nominal scales were used on the background questions. Ordinal scales (i.e. rank order without a fixed degree of difference between levels) were used to measure attitudes. Interval scales were used when measuring actual frequencies. During development and testing of the questionnaires, an open text box alternative was added to all response alternatives for refining purposes. These were, however, removed in the final survey.

## Results

Table 1 illustrates the implementation of alterations in the questionnaires to minimise measurement errors.

**Table 1. Progression in questionnaire formulation.**

| **Removed** | **Added** | **Comment / Other changes** |
| --- | --- | --- |
| *Subject-Matter Experts (SME), specialists in odontology in dogs and cats* | | |
|  | VHP:  At your workplace: Does a veterinarian *(in the nurse survey)* / veterinary nurse *(in the vet survey)* recommend that dog owners clean their dog's teeth? | Increased content validity |
|  | VHP: Do you follow up if dental home care for the dog is working? | Increased content validity |
|  | VHP: What do you consider to be the most common reason for dog owners to not brush their dog's teeth? | Increased content validity |
|  | DO: How important is it that your dog has good dental health? | Increased content validity |
|  | DO: Which of the following reasons for brushing the dog's teeth are the most important for you? | Increased content validity |
| *Reference group* | | |
| VHP: Question on intubation of the dog during dental cleaning |  | Potentially offensive (working formulation) |
| VHP: Question on the antibiotics used by the veterinarian |  | Potentially offensive (working formulation) |
| VHP: Employment? | VHP: Do you meet dogs in practice? | To simplify |
| DO: Question on chlorhexidine’s importance to dental health |  | Poor comprehensibility (working formulation) |
|  | DO: Are you a breeder? If so, do you inform puppy buyers about tooth brushing? | Increased content validity |
| DO and VHP: Gender: two separate response options “Other” respective “Prefer not to answer” | DO and VHP: Gender response option modified as one option: “Other / Prefer not to answer” | DO and VHP: “Other / Prefer not to answer” were set as one alternative instead of two separate alternatives, to avoid offensive reactions. |
|  |  | DO: Lay term always before technical term |
|  |  | DO: Offering condolences in introductory letter to owners whose dog was deceased. |
| *Cognitive interviews* | | |
|  |  | Specifications of unclear formulations (example in Fig. 2) |
| *Pilot* | | |
| VHP and DO: Name of commercial product removed |  | To avoid perceived product placement |
|  | DO: Question if calculus scraper was used by owner | Poor comprehensibility |
|  |  | Confirm function of logical jumps (Respondents were sent to different questions depending on their previous answers) |
|  |  | Specifications of unclear formulations (example in Fig. 2) |

DO denotes Dog Owner questionnaire, whereas VHP denotes Veterinary Health Practitioner questionnaire.

An example of the progress of question and response option formulation is shown in Fig. 2.

**Fig. 2. Example of progression of question and response options formulation, aiming to minimise measurement errors.**

**
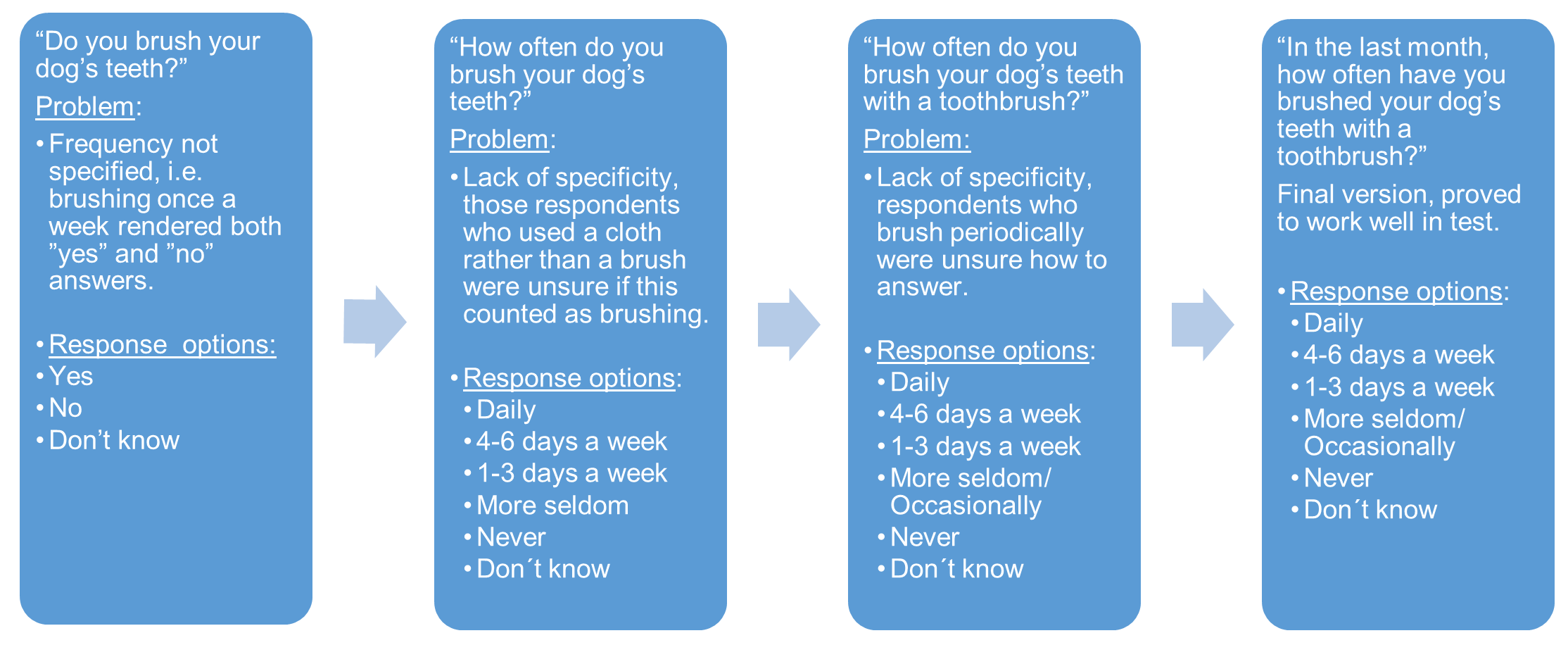
**

## Discussion

### Questionnaire construction

In general, measurement errors because of e.g. poor question wording or imperfect response scales are likely to be very common in questionnaire surveys (3). However, in this study, steps were taken to minimise this risk (Fig. 2). The ordering of the questions was chosen to minimise respondent influence and maintain interest (3). Therefore, questions on attitudes were placed before questions on practices. Similarly, to reduce non-completion in the questionnaire to the dog owners, background questions concerning the dog were placed first, whereas background questions concerning the respondents were placed towards the end of the questionnaire.

For some type of questions, vague quantifiers (“Sometimes”, “Often” and “Fairly”) were considered suitable. However, it should be noted that these words have different meanings in different contexts and for different people, and therefore do not reflect actual frequencies (6, 9). Fixed response alternatives were carefully formulated to reduce the risk of influencing respondents (Supporting information). Furthermore, fixed response alternatives were randomised to each respondent to avoid priming (3, 10). Different scales for response options were considered when constructing the different questionnaire items, and response options were also tested together with question formulations. Adjustments were made to find the ones best suited for the purpose (Fig. 2). Studies have shown that the most reliable and valid scales are of intermediate length, from 5-9 points (response options) (3). In the present study, to provide a balance between accuracy and user-friendliness, response options for ordinal or interval scales consisted of four or five levels, depending on whether a neutral or middle option was appropriate.

To manage respondent uncertainty, we chose to include a “don’t know” alternative, although a possible disadvantage with “don’t know” options can be that respondents use it without consideration. In fact, some data suggests that adding such an option does not improve measurement (3, 11-13). However, without a “don’t know” option, there is a risk of forcing the respondents to choose, even if they lack an opinion on the matter, which will lead to measurement errors. Therefore, we chose to include a “don’t know” alternative in accordance with what has been suggested by some authors (3, 14, 15).

Precautions were taken to avoid acquiescence bias, i.e. the tendency of a respondent to agree with a statement when in doubt, by careful wording of questions. Social desirability bias can also be problematic (3), and may lead to the owners and veterinary health practitioners reporting a false high degree of awareness and / or practice of dental home care. To avoid this as far as possible no judgmental formulations were used.

## References

1. Dillman DA, Smyth JD, Christian LM. Internet, Phone, Mail, and Mixed-Mode Surveys : The Tailored Design Method. New York, UNITED STATES: John Wiley & Sons, Incorporated; 2014.

2. Berntson E, Bernhard-Oettel C, Hellgren J, Näswall K, Sverke M. Enkätmetodik. Stockholm: Natur och kultur; 2016 2016. 309 p.

3. Marsden PV, Wright JD. Handbook of Survey Research: Emerald; 2010.

4. Edwards PJ, Roberts I, Clarke MJ, Diguiseppi C, Wentz R, Kwan I, et al. Methods to increase response to postal and electronic questionnaires. Cochrane database of systematic reviews (Online). 2009(3).

5. Sijtsma K. On the Use, the Misuse, and the Very Limited Usefulness of Cronbach’s Alpha. Psychometrika. 2009;74(1):107-20.

6. Tourangeau R, Rips LJ, Rasinski K. The Psychology of Survey Response. Cambridge: Cambridge University Press; 2000.

7. Ejlertsson G. Enkäten i praktiken : en handbok i enkätmetodik. 2 ed. Lund: Studentlitteratur; 2005 2005. 157 p.

8. RFSL. To Ask About Gender and Trans in Surveys 2016 [Available from: <https://www.rfsl.se/en/lgbtq-facts/to-ask-about-gender-and-trans-in-surveys/>.

9. Cliff N. Adverbs as multipliers. Psychol Rev. 1959;66(1):27.

10. Bradburn NM, Sudman S, Wansink B. Asking questions: the definitive guide to questionnaire design--for market research, political polls, and social and health questionnaires: John Wiley & Sons; 2004.

11. Gilljam M, Granberg D. Should We Take Don't Know for an Answer? The Public Opinion Quarterly. 1993;57(3):348-57.

12. Poe GS, Seeman I, McLaughlin J, Mehl E, Dietz M. “Don't know” boxes in factual questions in a mail questionnaire. Effects in level and quality of response. Public Opin Q. 1988;52(2):212-22.

13. Krosnick JA, Holbrook AL, Berent MK, Carson RT, Michael Hanemann W, Kopp RJ, et al. The Impact of "No Opinion" Response Options on Data Quality: Non-Attitude Reduction or an Invitation to Satisfice?*. Public Opin Q. 2002;66(3):371-403.

14. Payne SL. Case Study in Question Complexity. Public Opin Q. 1949;13(4):653-8.

15. Vaillancourt PM. Stability of Children's Survey Responses. The Public Opinion Quarterly. 1973;37(3):373-87.
